# Cretaceous Blind Snake from Brazil Fills Major Gap in Snake Evolution

**DOI:** 10.1016/j.isci.2020.101834

**Published:** 2020-11-20

**Authors:** Thiago Schineider Fachini, Silvio Onary, Alessandro Palci, Michael S.Y. Lee, Mario Bronzati, Annie Schmaltz Hsiou

**Affiliations:** 1 Laboratório de Paleontologia, Faculdade de Filosofia Ciências e Letras de Ribeirão Preto, Universidade de São Paulo, Ribeirão Preto, São Paulo, Brazil; 2 College of Science and Engineering, Flinders University, Adelaide, SA 5042, Australia; 3 South Australian Museum, North Terrace, Adelaide, SA 5000, Australia; 4 Laboratório de Evolução e Biologia Integrativa, Faculdade de Filosofia Ciências e Letras de Ribeirão Preto, Universidade de São Paulo, Ribeirão Preto, São Paulo, Brazil

**Keywords:** Paleontology, Animals, Systematics, Phylogenetics, Evolutionary History, Paleobiology

## Abstract

Blind snakes (Scolecophidia) are minute cryptic snakes that diverged at the base of the evolutionary radiation of modern snakes. They have a scant fossil record, which dates back to the Upper Paleocene-Lower Eocene (∼56 Ma); this late appearance conflicts with molecular evidence, which suggests a much older origin for the group (during the Mesozoic: 160–125 Ma). Here we report a typhlopoid blind snake from the Late Cretaceous of Brazil, *Boipeba tayasuensis* gen. et sp. nov, which extends the scolecophidian fossil record into the Mesozoic and reduces the fossil gap predicted by molecular data. The new species is estimated to have been over 1 m long, much larger than typical modern scolecophidians (<30 cm). This finding sheds light on the early evolution of blind snakes, supports the hypothesis of a Gondwanan origin for the Typhlopoidea, and indicates that early scolecophidians had large body size, and only later underwent miniaturization.

## Highlights

• *Boipeba tayasuensis* is the oldest fossil blind snake from the Late Cretaceous of Brazil
• A new phylogenetic analysis places the taxon within living typhlopoids
• *Boipeba* is estimated to be ∼1 m in length, larger than any living blind snake
• The small body size of extant blind snakes is due to subsequent miniaturization

## Graphical Abstract

**Figure:**
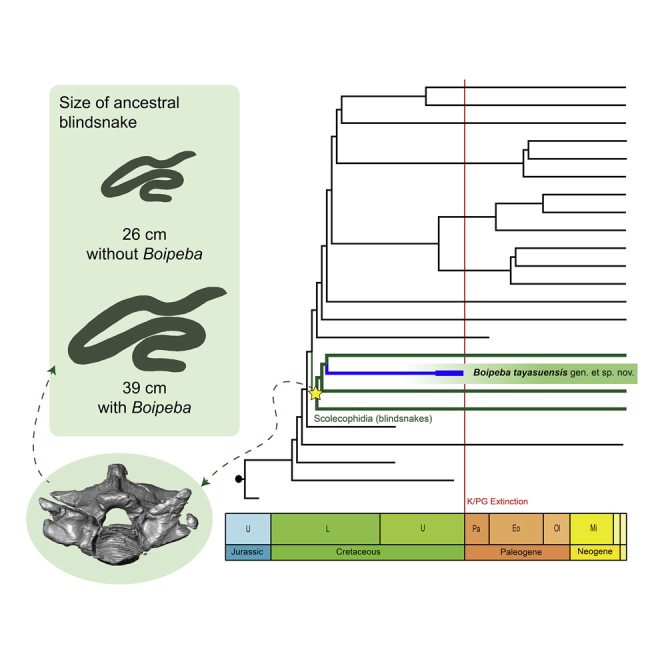

## Introduction

Snakes comprise one of the most successful radiations of land vertebrates, with over 3,800 living species (Uetz et al., 2020). With ∼620 species, blind snakes (Scolecophidia) represent a significant portion of snake diversity (Uetz et al., 2020). They consist of small worm-like snakes, generally less than 30 cm in total length (TL) (Hedges, 2008; Feldman et al., 2016), with adaptations linked to their burrowing lifestyle such as a small subterminal mouth, uniquely modified jaws, reduced eyes covered by a large scale, and a cylindrical body with similar cranial and caudal ends (Cundall and Irish, 2008; Hsiang et al., 2015).

The origin of blind snakes is unclear. Their morphology includes a mixture of seemingly primitive lizard-like features and highly specialized characters (List, 1966), and there is disagreement between morphological and molecular phylogenetic analyses with regard to their phylogenetic position and monophyly (Zheng and Wiens, 2016; Figueroa et al., 2016; Garberoglio et al., 2019a; Caldwell 2019). Furthermore, while most recent molecular analyses agree on the non-monophyly of scolecophidians (but see Singhal et al., 2020), they still disagree on their branching order, with anomalepidids placed either in a more basal or more derived position relative to the other blind snake lineages (Leptotyphlopidae and Typhlopoidea) (Zheng and Wiens, 2016; Figueroa et al., 2016; Miralles et al., 2018). Regardless of this inconsistency, molecular analyses agree that blind snakes are basal to other living snakes, and thus have very ancient origins, sometime between the Upper Jurassic and the Lower Cretaceous (160–125 Ma) (Zheng and Wiens, 2016; Vidal et al., 2010; Burbrink et al., 2020) in Gondwana (Vidal et al., 2010; Pyron and Wallach, 2014). However, the oldest occurrence of scolecophidians in the fossil record currently dates back only to the Upper Paleocene-Lower Eocene (c. 56 Ma) of Europe and northern Africa (Rage, 1984; Augé and Rage, 2006), implying the existence of a large fossil gap. The basal position of blindsnakes with respect to other living snakes also means they provide crucial information on the evolution of living snakes (e.g., da Silva et al., 2018).

Here we report on a giant fossil scolecophidian found in Late Cretaceous sediments from Brazil. This finding sheds new light on the origin of blind snakes, bridging the gap between molecular and paleontological evidence (Zheng and Wiens, 2016; Vidal et al., 2010; Pyron and Wallach, 2014; Burbrink et al., 2020). Furthermore, the new fossil also provides insights into scolecophidian body size evolution, showing that extreme miniaturization is likely a derived trait within these highly specialized snakes, and thus small size cannot be assumed to characterize the ancestral blind snake or the most recent common ancestor of modern (crown) snakes in general.

## Results

### Systematic Palaeontology

Squamata Oppel, 1811

Ophidia Brongniart, 1800

Scolecophidia Duméril and Bibron, 1844

**Figure 1:**
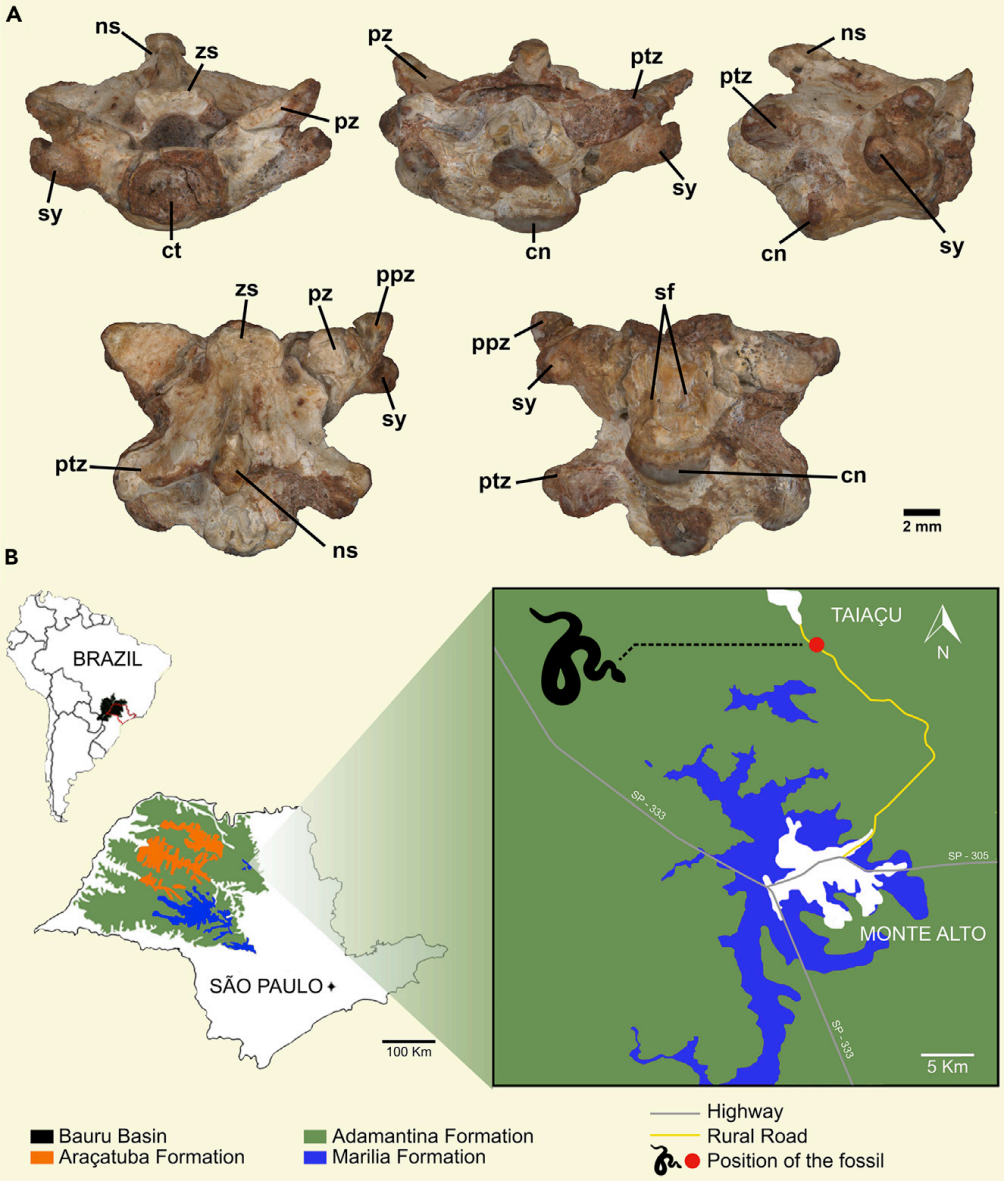
Holotype of *Boipeba tayasuensis* (A) MPMA 16-0008-08, isolated precloacal vertebra in (upper row) anterior, posterior, and lateral views, respectively, and (lower row) dorsal, and ventral views, respectively. (B) Geographical and geological map showing the type locality where the fossil material was recovered. Abbreviations: cn., condyle; ct., cotyle; ns., neural spine; ptz., postzygapophysis; ppz., prezygapophyseal accessory processes; pz., prezygapophysis.; sf., subcentral foramina; sy., synapophysis; zs., zygosphene.

**Figure 2:**
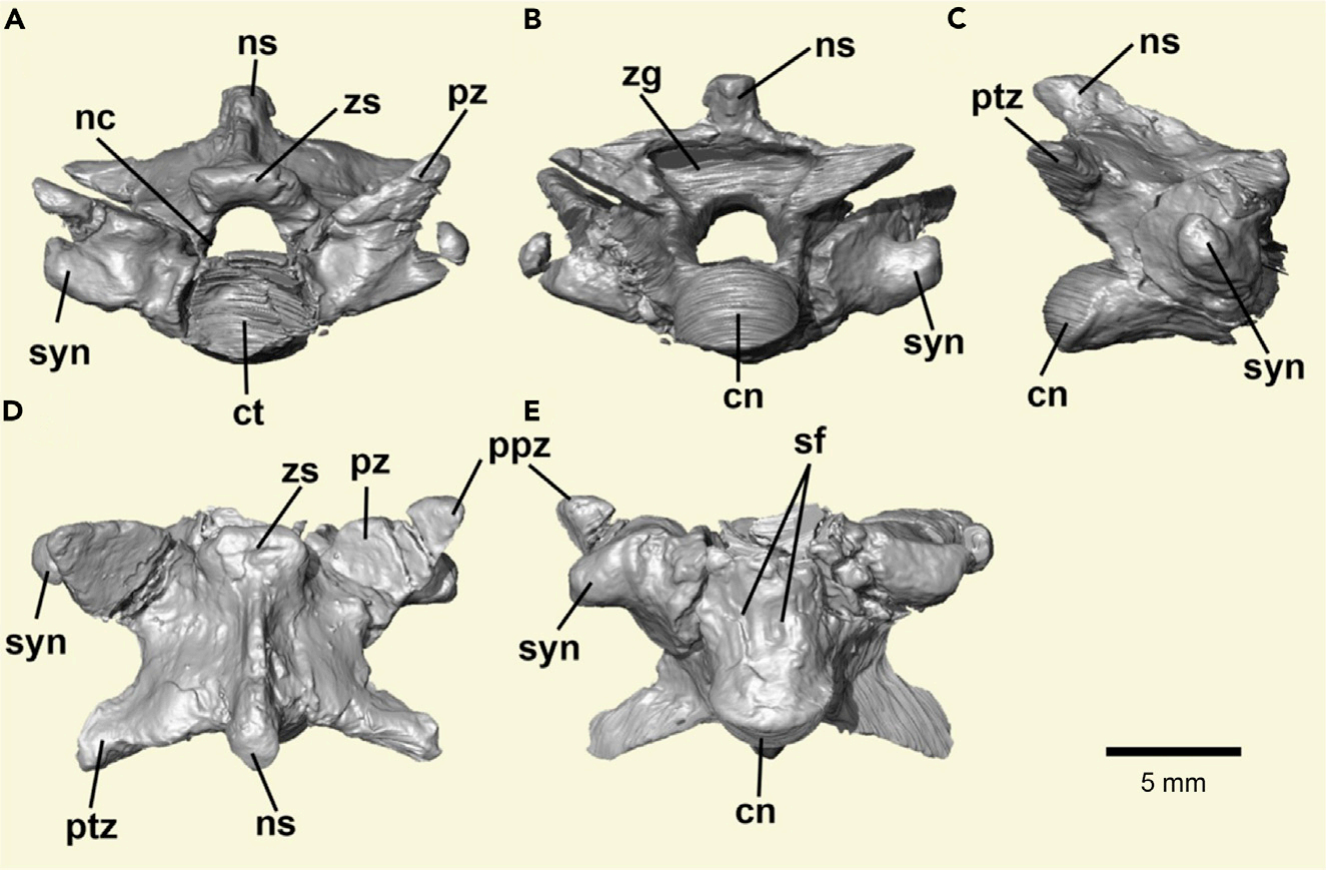
Three-Dimensional Reconstruction of *Boipeba tayasuensis* (A–E) MPMA 16-0008-08, isolated precloacal vertebra in (A) anterior, (B) posterior, (C) lateral, (D) dorsal, and (E) ventral views. Cn, condyle; ct., cotyle; nc, neural canal; ns., neural spine; ptz., postzygapophysis; ppz., prezygapophyseal accessory processes; pz., prezygapophysis.; sf., subcentral foramina; syn., synapophysis; zg, zygantrum; zs., zygosphene.

*Boipeba tayasuensis* gen. et sp. nov.

(Figures 1, 2, S1, and S2)

#### Etymology

Generic epithet comes from the combination of the Brazilian native language Tupi-Guarani, “boi” meaning snake, and “peba” meaning flattened, in reference to the shape of the vertebrae. The species epithet “tayasuensis” derives from the type locality where the fossil was found, Taiaçu municipality, São Paulo, Brazil.

#### Holotype

MPMA 16-0008-08, a single precloacal vertebra with partial successive vertebra (MPMA = Museu de Paleontologia Prof. Antônio Celso de Arruda Campos, Monte Alto, São Paulo State, Brazil).

#### Locality and Horizon

The fossil comes from a rural road between the municipalities of Monte Alto and Taiaçu, in the Northwest of the state of São Paulo, Brazil (Figure 1B) (S21° O9′53.9″/W48°29′54.0″). The outcrop bearing the new material is a rich fossiliferous locality that preserves an abundant fauna including crocodyliforms (Carvalho et al., 2007; Iori and de Souza Carvalho, 2009; Iori and Carvalho, 2011; Iori and Garcia, 2012; Iori et al., 2013; Iori and Campos, 2017), testudines (Ferreira et al., 2018), and dinosaurs such as sauropods and non-avian theropods (Méndez et al., 2014; Santucci and Arruda-Campos, 2011; Tavares et al., 2014). The sediments in the outcrop consist of the typical reddish muddy sandstones of the Adamantina Formation (Bauru Basin) found in the Monte Alto region (Batezelli, 2017). The time span of the Adamantina Formation has been the matter of a long debate, with some works estimating a Turonian-Santonian age (Dias-Brito et al., 2001), whereas others suggest a younger age, between Campanian-Maastrichtian (Batezelli, 2017; Gobbo-Rodrigues et al., 1999), or a broader range, Cenomanian-Maastrichtian (Menegazzo et al., 2016). There are no integrative absolute date studies for the Adamantina Formation to specify the age correlation among the different fossiliferous localities found in the Bauru Group. Recently, the first high-precision U-Pb geochronology study has shown a post-Turonian maximal age (**≤**87.8 Ma) for the type stratum of *Brasilestes stardusti* (Castro et al., 2018), which is overlain by the dinosaur-bearing Marília Formation; this age thus constrains the maximum age of the Adamantina Formation at the *Boipeba* site. The minimum age is not well constrained, but presence of non-avian dinosaurs in higher beds implies an age pre-dating the Cretaceous-Paleogene (K/Pg) boundary (66 Ma) (Batezelli, 2017; Menegazzo et al., 2016).

#### Diagnosis

Medium-sized snake vertebra (∼7-mm-anteroposteriorly-long centrum) distinguished from all other ophidians in possessing the following unique combination of vertebral features: dorsoventrally compressed vertebra having oval cotyle and condyle; zygosphene with straight anterior margin; prezygapophyseal articular facets with high angle of inclination (∼25°) above the horizontal plane; presence of elongated prezygapophyseal accessory processes; undivided synapophyses (i.e., no distinct diapophyseal and parapophyseal facets) mediolaterally expanded to the level of the prezygapophyseal articular facets; synapophyses located above the ventral margin of the cotyle; low neural spine slanting posteriorly; shallowly concave posterior margin of neural arch; cylindrical centrum lacking parasagittal ridges and hemal keel (at least in middle/posterior trunk vertebrae, unknown in anterior vertebrae); lack of paracotylar foramina; lack of parazygantral foramina; weak precondylar constriction; and asymmetrical subcentral foramina.

### Description

The holotype consists of an isolated vertebra articulated with the anterior region of a fragmentary following vertebra. It is likely to belong to the middle or posterior precloacal region due to the absence of structures such as hypapophyses, lymphapophyses, pleurapophyses, or hemapophyses. The vertebra is three-dimensionally preserved. The neural arch is mediolaterally expanded and dorsoventrally compressed. The zygantrum is deep and has a pair of foramina inside. In dorsal view the neural arch displays a shallowly concave posterior embayment. In ventral view, the centrum is cylindrical, completely smooth (i.e., hemal keel absent), with a weak precondylar constriction. On the ventral surface of the centrum there is a pair of asymmetrical subcentral foramina, where the left foramen is small but distinct, whereas on the right side only a broad shallow fossa is visible, and the foramen has not fully developed through the bone (Figures 2, S1, and S2), a condition found in some other living and fossil scolecophidians (Mead, 2013).

The neural spine is low, posterodorsally inclined, and in dorsal view, extends longitudinally from the posterior region of the zygosphene roof to slightly beyond the posterior embayment of the neural arch. The zygosphene is robust and partly eroded in the three-dimensionally preserved main specimen; however, in the fragmentary successional vertebra it is well preserved and characterized by a rectilinear anterior margin (Figure S1). The neural canal is vaulted.

The prezygapophyseal articular facets are broad, subtriangular in shape, and inclined above the horizontal about 25° (average between left and right sides). Long prezygapophyseal accessory processes are present. The process on the left side is partially worn, but the one on the right is complete (Figures 1 and 2). These processes stand out quite distinctly when two vertebrae (one being a digital replica) are placed in articulation (Figure S2). The synapophyses are undivided (i.e., no distinct para- and diapophyses), extend laterally to the level of the prezygapophyseal articular facets, and are placed dorsal to the ventral margin of the cotyle. Both cotyle and condyle are oval (i.e., dorsoventrally compressed) in anteroposterior view.

#### Systematic Comparisons to Other Ophidians

**Figure 3:**
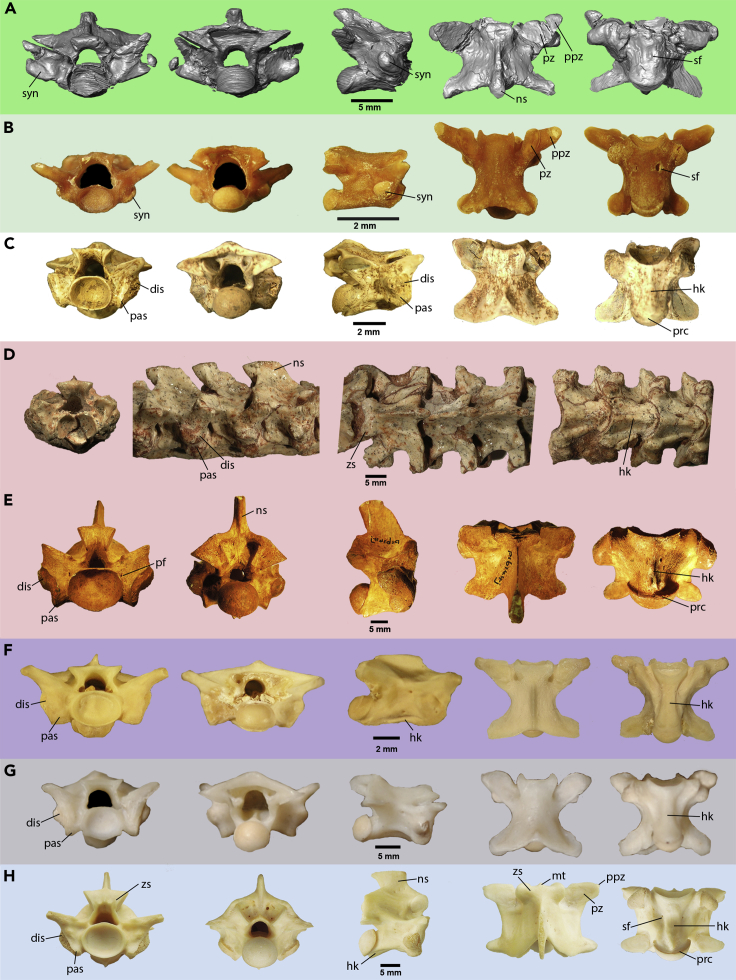
Vertebral Comparisons among *Boipeba tayasuensis* and Selected Ophidian Specimens Background colors match the groups in Figure 4. (A) *Boipeba tayasuensis*, 3D model surface rendering (MPMA 16-0008-08). (B) *Afrotyphlops punctatus* (USNM 320704). (C) *Coniophis precedens* (UALVP unnumbered specimen). (D) *Dinilysia patagonica* (MACN-RN unnumbered specimen). (E) *Wonambi naracoortensis* (SAMA P16168). (F) *Anilius scytale* (MCZ, 19537). (G) *Cylindrophis ruffus* (MNHN, 1869 771). (H) *Simalia amethistina* SAMA R2605. With the only exception of *Dinilysia*, views from right to left are in anterior, posterior, right lateral, dorsal, and ventral views; anterior, right lateral, dorsal, and ventral for *Dinilysia*. Lateral view was mirrored in *Coniophis* and *Wonambi* for ease of comparison. Abbreviations: dis., diapophyseal articular facet of synapophyses; hk., hemal keel; mt., median tubercle; ns., neural spine; pas. parapophyseal articular facet of synapophyses; pcr., precondylar constriction; pf., paracotylar foramen; ppz., prezygapophyseal processes; pz., prezygapophysis; sf., subcentral foramen; syn., synapophysis; zs., zygosphene.

Despite the apparent conservative morphology of snake vertebrae, a set of anatomical features can be used to identify them at least to some broad taxonomic level when found in isolation (Rage, 1984). *Boipeba* retains a mix of plesiomorphic features observed in stem snakes together with apomorphic traits typical of some representatives of the crown-group (modern snakes), the clade stemming from the most recent common ancestor of all living snakes (Figures 2 and 3; Table 1). We show below that *Boipeba* is most similar to blind snakes (scoleophidians) and distinct from all other snakes.

**Table 1:** Comparative Vertebral Traits between Selected Ophidian Taxa

Vertebral Traits	*Boipeba tayassuensis*	Scolecophidia (*Afrotyphlops punctatus*)	*Coniophis precedens*	*Dinilysia*	Madtsoiidae (*Wonambi naracoortensis*)	*Anilius scytale*	Macrostomata (*Simalia amethistina*)
Confluent synapophyses	Present∗	Present∗	Absent	Absent	Absent	Absent	Absent
Synapophyses in the same level of prezygapophyseal articular facets	Present	Absent	Absent	Absent	Present	Absent	Absent
Presence of long prezygapophyseal accessory processes	Present	Present	Absent	Absent	Absent	Present	Absent
Dorsoventrally flattened neural arch	Present	Present	Present	Absent	Absent	Present	Absent
Shallow posterodorsal margin of the neural arch	Present	Present	Present	Present	Absent	Present	Absent
Low neural spine	Present	Present	Present	Absent	Absent	Present	Absent
Cylindrical: centrum	Present	Present	Present	Absent	Absent	Absent	Absent
Smooth ventral margin of the centrum	Present∗	Present∗	Absent	Absent	Absent	Absent	Absent
Ellipsoidal dorsoventrally compressed cotyle	Present	Present	Variable	Variable	Absent	Absent	Absent
Asymmetrical subcentral foramina	Present	Present	Absent	Present	Absent	Present	Absent

∗ Morphological traits exclusively shared between *Boipeba tayasuensis* and scolecophidians.

Among all the extant and extinct snakes known, *Boipeba tayasuensis* (Figures 2 and 3A) shows a combination of vertebral features that is only observed in modern scolecophidians, most notably in typhlopoids *sensu* Pyron and Wallach (2014) (Figure 3B). The vertebral features that are shared between *Boipeba* and Scolecophidia (Figures 3A and 3B; Table 1) include (1) dorsoventrally flattened vertebra, (2) absence of median notch in the posterior border of the neural arch, (3) narrow and cylindrical centrum, (4) absence of hemal keel and/or median ventral prominence between the cotyle and condyle, (5) presence of asymmetrical subcentral foramina, (6) weakly developed precondylar constriction, (7) cotyle and condyle oval in anteroposterior view, (8) the presence of well-developed prezygapophyseal processes, and (9) undivided synapophyses with no distinction between the para- and diapophyseal articular facets (Rage, 1984). Additionally, modern typhlopoids, exemplified by *Afrotyphlops punctatus* (Figure 3B), exclusively share with *Boipeba* the high position of the synapophyses, which are located dorsal to the ventral margin of the cotyle. Despite the overall similarity, *Boipeba* differs from modern scolecophidians in having a wider and shorter vertebra, synapophyses that extend further laterally, and a taller (though still relatively low) neural spine.

Despite its clear scolecophidian similarities, *Boipeba* also has some features in common with other basal fossil snakes. Early stem snakes, informally termed “parviraptorids” (Caldwell et al., 2015), share with *Boipeba* the presence of a smooth ventral margin on a cylindrical centrum and the weakly developed precondylar constriction. However, unlike *Boipeba*, “parviraptorids” display very small zygantra and zygosphenes, rounded cotyles and condyles, tall neural spines, absence of prezygapophyseal accessory processes, very steep pre- and postzygapophyseal facets (∼40 above horizontal), and synapophyses subdivided into para- and diapophyseal facets (Caldwell et al., 2015). Madtsoiid snakes (e.g., *Wonambi*, Figure 3E), as well as the South American Late Cretaceous stem-snakes *Dinilysia* (Figure 3D, Table 1) and the hind-limbed *Najash* share with *Boipeba* features like the synapophyses extending approximately to the same level of the lateral margin of the prezygapophyseal articular facets, and a shallowly concave posterior margin of the neural arch embayment. However, these stem-snakes differ from *Boipeba* in possessing a well-developed neural spine, rounded cotyle and condyle, prominent hemal keel in a subtriangular centrum with a marked constriction, presence of paired parazygantral foramina (only in madtsoiids and *Najash*), synapophyses with division between the para- and diapophyseal facets, absence of prezygapophyseal accessory processes, and the occurrence of parasagittal and arqual ridges in the neural arch (Zaher et al., 2009; Laduke et al., 2010; Garberoglio et al., 2019a).

The fossil snake *Xiaophis myanmarensis* (Xing et al., 2018), albeit most likely a neonate, presents mid-trunk vertebrae that are remarkably similar to those of *Dinilysia* in general proportions. *Xiaophis* shares with *Boipeba* the presence of low and posteriorly tilted neural spines but can be readily distinguished by the presence (in *Xiaophis*) of synapophyses that are subdivided into para- and diapophyseal facets, distinct hemal keels, and smaller prezygapophyseal processes. The last feature may be due to the early ontogenetic stage of the snake, whereas the other two features cannot be so readily explained.

The only unambiguous Cretaceous ophidian record from the Cenomanian of Brazil, the stem-snake *Seismophis septentrionalis* (Hsiou et al., 2014) shares with *Boipeba* the vaulted neural canal morphology, the relatively low neural arch (in posterior view), and the weakly developed neural spine, but is clearly distinguished from the latter in possessing a flattened hemal keel, presence of parazygantral foramina, rounded cotyle and condyle, and marked parasagittal ridges.

The long-bodied squamate *Tetrapodophis* from the Early Cretaceous of Brazil, which was described as a stem-snake (Martill et al., 2015; but see Caldwell et al., 2016, Paparella et al., 2018, and Caldwell, 2009 for an alternative interpretation), shares with *Boipeba* features such as the low neural spine and neural arch, but differs from the latter in possessing a deep V-shaped posterior margin of the neural arch, divided synapophyses, and the presence of well-defined hemal keels and subcentral fossae. In contrast, *Boipeba* possesses a shallow posterodorsal embayment of the neural arch, undivided synapophyses, and a smooth ventral surface of the centrum.

The Cretaceous (Cenomanian) marine Tethyan Pachyophiidae are characterized by pachyostotic vertebrae, a diagnostic feature not observed in *Boipeba*. Moreover, pachyophiids lack prezygapophyseal processes, which are well developed in *Boipeba*.

Another aquatic fossil snake from the Upper Cretaceous (Cenomanian) of Venezuela, *Lunaophis aquaticus* (Albino et al., 2016), shares with pachyophiids the presence of pachyostosis and the lack of prezygapophyseal processes and has a distinct hemal keel and an elongate subtriangular centrum in ventral view. Thus, *Lunaophis* can also be readily distinguished from *Boipeba*.

The Cretaceous fossil snake *Coniophis precedens* (Figure 3H) shares with modern scolecophidians (Figure 3C) and *Boipeba* (Figures 2 and 3A), features such as the dorsoventrally compressed vertebra, oval cotyle and condyle, relatively low neural spine, narrow centrum, and weak precondylar constriction. However, the absence of prezygapophyseal processes and a flattened hemal keel surrounded by subcentral groves make this extinct snake morphologically different from both scolecophidians and *Boipeba*.

Among alethinophidians, i.e., modern (crown) snakes apart from blind snakes, *Boipeba* shares some features with members of the “Amerophidia” (i.e., *Anilius* + *Tropidophis*) and Uropeltoidea (Figures 2F and 2G), like the dorsoventrally compressed vertebral morphology, a shallowly concave posterior neural arch margin (in dorsal view), the relative steep inclination of the prezygapophyseal articular facets, the presence of well-developed prezygapophyseal accessory processes, zygosphene morphology characterized by a straight anterior edge, and a low neural spine (only in *Anilius*). On the other hand, both amerophidians and uropeltoids are distinct from *Boipeba* in displaying division between the articular facets of the synapophyses, distinctly trifoliate neural canal morphology, flattened hemal keel delimited by subcentral grooves, rounded cotyle and condyle, and subtriangular centrum with marked precondylar constriction. Furthermore, *Boipeba* differs from *Cylindrophis* (Figure 2F) due to the presence of a neural spine (absent in the latter) and from *Anilius* (Figure 2G) in having the neural spine that extends posteriorly beyond the posterior margin of the neural arch.

The vertebrae of afrophidian snakes (i.e., Henophidia + Caenophidia) differ from those of *Boipeba* in many respects. In general, Henophidia (e.g., boids and pythonids like *Simalia amethistina*; Figure 2H) sharply differ from the Cretaceous fossil due to a well-developed neural spine, broad and vaulted neural arch (in posterior view) with marked parasagittal ridges and a deep posterodorsal notch, massive zygosphene with median tubercle (in some species), synapophyses subdivided in para- and diapophyses, rounded cotyle and condyle, prezygapophyseal accessory processes reduced to a small pyramidal projection, variable presence of paracotylar and neural arch foramina (sensu Onary and Hsiou, 2018), weak interzygapophyseal constriction, pre- and postzygapophyseal facets that are typically inclined between 0° and 15° above horizontal (steeply inclined in *Boipeba*, ∼25°), and a broad subtriangular centrum with prominent hemal keel and strong precondylar constriction. *Boipeba* shares with members of the Caenophidia (e.g., Colubroidea) the presence of elongated prezygapophyseal processes. However, colubroids have lightly built, elongated vertebrae, synapophyses subdivided into para- and diapophyses, frequent presence of paracotylar foramina, low inclination of the pre- and postzygapophyseal facets, and retain hypapophyses throughout the vertebral column (Rage, 1984).

#### Phylogeny

**Figure 4:**
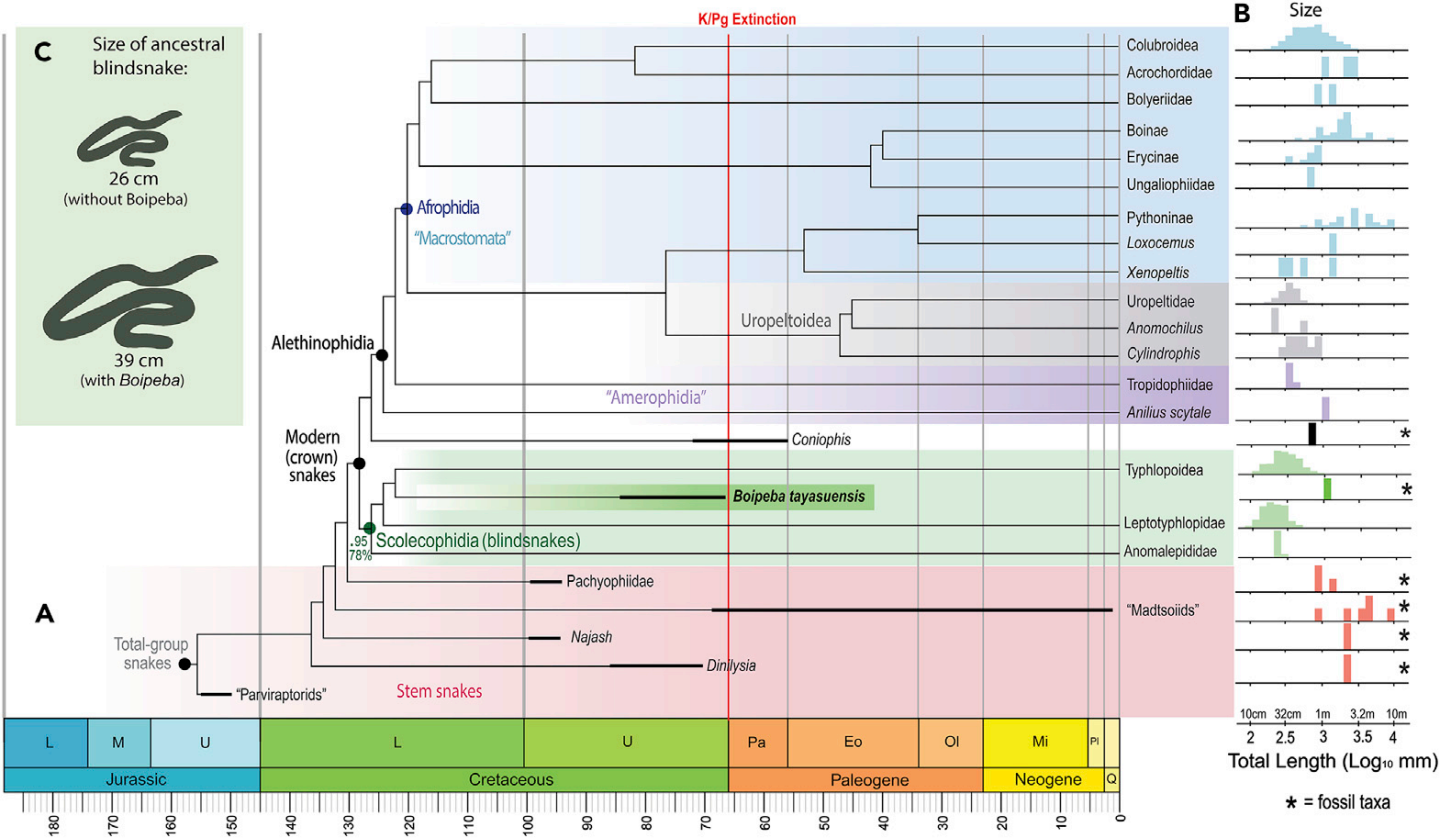
*Boipeba* and the Evolution of Snakes Taxon shading colors match the scheme in Figure 3. (A) Phylogenetic relationships of the giant fossil blind snake *Boipeba* and other major snake lineages, based on Bayesian and parsimony analyses of morphology and DNA (tree topology as in Figure S3A; numbers at blind snake clade are Bayesian posterior and parsimony bootstrap support. Divergence dates for living snakes are based on molecular dates (for compatible clades) in Zheng and Wiens (2016); bold lines indicate stratigraphic range or uncertainty for fossil taxa. Quotes denote non-monophyletic taxon names. (B) Size distribution of all species for each major living snakes lineage and important fossil taxa, on a log scale; note *Boipeba* is larger than living blind snakes. (C) *Boipeba* greatly increases the size estimate for the most recent common ancestor of living blind snakes (see also Figure S9).

All vertebral features present in *Boipeba* are fully consistent with what is found in Serpentes, and in particular modern and fossil scolecophidians (List, 1966; Rage, 1984; Mead, 2013); no other vertebrate group is remotely similar. We thus tested the phylogenetic relationships of *Boipeba* by inserting it into a morphological data matrix for the major lineages of living and fossil snakes, expanded from a recent study (Garberoglio et al., 2019b see Methods). *Boipeba* could be scored for 29 vertebral characters out of the 253 morphological characters (see Transparent Methods section). This morphological matrix was analyzed alone, and in combination with DNA data (for living taxa) consisting of 18,753 base pairs from 17 genes (7 mitochondrial and 10 nuclear) from Tonini et al. (2016). Analyses used maximum parsimony (PAUP, Swofford and Sullivan, 2003, and TNT, Goloboff et al., 2008) and undated Bayesian (MrBayes, Ronquist et al., 2012) optimality criteria (see Transparent Methods). Initial analyses with all 37 terminal taxa had support deflated due to “wildcard” taxa with large amounts of missing data, so additional analyses included only the 33 most complete taxa (excluding 4 taxa with >90% missing data). Regardless of the optimality criteria employed or the character/taxon sampling (morphology alone or combined molecular and morphological data, all taxa or fragmentary/contentious taxa excluded), *Boipeba tayasuensis* always emerged unambiguously with scolecophidian affinities, and thus within crown snakes (see Figures 4A and S3–S5). For instance, in the combined morphological and molecular analyses of all 37 taxa (excluding *Tetrapodophis*), support uniting *Boipeba* with all scolecophidians was 72% (parsimony partitioned bootstrap) and 0.99 (Bayesian posterior probability). Within scolecophidians, *Boipeba* was the sister taxon to typhlopoids (here represented by *Typhlops*), but this relationship was not robust (51% bootstrap, 0.65 posterior probability).

There are three unambiguous morphological synapomorphies uniting *Boipeba* with Scolecophidia: the presence of a confluent synapophysis (character 116), the presence of elongated prezygapophyseal accessory processes (character 117), and a smooth ventral margin of the centrum (character 121). Furthermore, the position of the synapophyses placed dorsal to the ventral margin of the cotyle (character 249) is a synapomorphy shared between *Boipeba* and Typhlopoidea. Thus, despite being represented only by vertebral characters, the analyses support a scolecophidian affinity of *Boipeba.*

With regard to the relationships among other snakes, our preferred topology (Figure 4A) is similar to that presented in Garberoglio et al. (2019b), where “parviraptorids” are the earliest diverging stem snakes, followed by *Dinilysia*, *Najash*, the paraphyletic assemblage “madtsoiids,” and the marine Pachyophiidae; the last taxon is the sister group to crown-snakes. The North American Cretaceous fossil *Coniophis precedens* was weakly recovered as the sister taxon to the Alethinophidia, *contra* Longrich et al., (2012a), who found it to be the sister taxon to all extant snakes (crown-snakes). *Tetrapodophis* (Martill et al., 2015) was initially excluded from our phylogenetic analyses of snakes due to ongoing debate over its status as a snake (Caldwell et al., 2016; Paparella et al., 2018; Caldwell, 2019). However, we also repeated all analyses adding *Tetrapodophis* to the snake ingroup, as a candidate early snake (Martill et al., 2015). The resulting topologies still retrieved *Boipeba* in the same position within Scolecophidia, and with slightly increased parsimony support (76% partitioned bootstrap) and similar Bayesian support (0.99 PP) (Figures S6–S8).

#### Size estimate of *Boipeba* and the Ancestral Scolecophidian

The fossil snake *Boipeba tayasuensis* has a centrum length (CL) of 6.8 mm (measured along the ventral margin), which is very large compared with the minute vertebrae typically found in extant scolecophidians (List, 1966) (see also Data S1). We estimated the total length of *Boipeba* by using the relationship between total length and vertebral length found in extant scolecophidians (see Methods). We obtained an average estimate of total length of 1.1 m for *Boipeba* (Figure 4B and Data S1). When compared with other fossil snakes, *Boipeba* is similar in length to pachyophiids and *Najash* (∼1 m), about half the length of *Dinilysia* (∼2 m), and is considerably shorter than most “madtsoiids,” which have an average total length of about 4.4 m (Figure 4B). The fossil snake *Coniophis* has an estimated TL of about 0.7 m (Longrich et al., 2012a), which is shorter than the estimated TL of *Boipeba* (1.1 m).

Among basal alethinophidians, *Boipeba* is similar in length to *Anilius*, whereas double the length of most tropidophiids and uropeltids (Figure 4B). Afrophidian snakes (i.e., Henophidia + Caenophidia) exhibit a wide range of sizes within each clade. Colubroid caenophidians have an average size that is smaller (∼0.8 m) than that of *Boipeba* (Figure 4B), whereas some henophidian groups such as Boinae and Pythoninae, which include the largest extant snakes, are on average about two to three times longer (average TL of Pythoninae ∼2 m; average TL of Boinae ∼3 m; Figure 4B) (Feldman et al., 2016).

*Boipeba* therefore represents an exceptionally large extinct scolecophidian, four times longer than the average anomalepidid (∼0.25 m), about five times the average leptotyphlopid (∼0.20 m), and nearly three and a half times the average typhlopoid (∼0.31 m). It is much closer in size to typical alethinophidians, as well as to most basal fossil snakes (Figure 4B).

The size and phylogenetic position of *Boipeba* sheds substantial light on the evolution of body size within scolecophidian snakes. We reconstructed the size (TL) of the ancestral blind snake using a dated, well-sampled (98 extant species) phylogeny of Scolecophidia (Zheng and Wiens, 2016) and size (TL) data from Feldman et al. (2016). *Boipeba* was inserted into this phylogeny midway along the relevant branch (sister taxon to typhlopoids), and size estimates for all ancestral nodes were obtained using parsimony/likelihood methods (see Methods). When *Boipeba* is included, the ancestral scolecophidian is estimated to have had a total length of about 0.39 m (see Methods, Figures 4C and S9), whereas the analysis using only living scolecophidian taxa retrieved an estimated ancestral body length of only 0.26 m (see Transparent Methods section). This 1.5-fold length increase would translate to a ∼3-fold mass increase assuming isometry (1.5^3^ = 3.375). The TL estimate for the ancestor of living typhlopoids is similarly affected, with an estimate of 0.40 m when the fossil is included in the analysis versus an estimate of 0.30 m when it is excluded (see Transparent Methods section).

## Discussion

**Figure 5:**
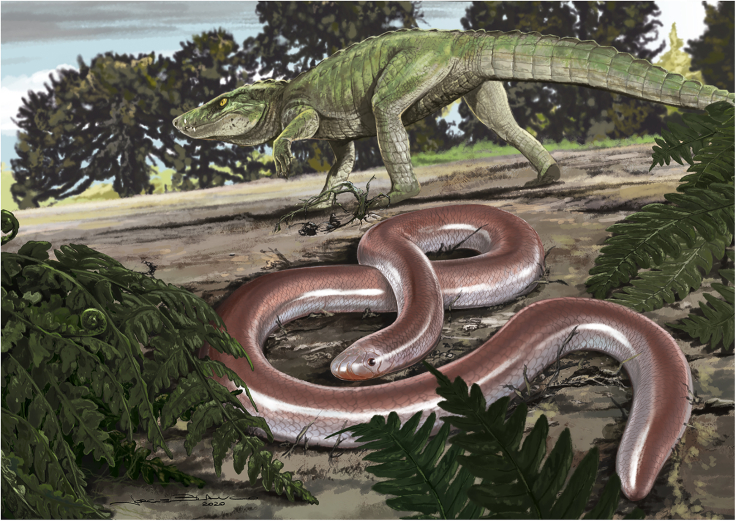
Life Reconstruction of *Boipeba tayasuensis* This large Cretaceous blind snake inhabited the arid palaeoenviroment of the Bauru Basin, Brazil, alongside titanosaur sauropods, theropods, and terrestrial crocodiles such as *Montealtosuchus* (Mesoeucrocodylia, Peirosauridae). The latter was found in the same outcrop as *Boipeba*. Reconstruction by Jorge Blanco.

*Boipeba tayasuensis* sheds light on important aspects of the early evolution of blind snakes in terms of timing, geographic origin, and body size. Before the discovery of *Boipeba,* the oldest scolecophidians were known from the Eocene of Europe (Rage, 1984) and the Paleocene of Morocco (Augé and Rage, 2006). Thus, *Boipeba* provides the first evidence for their presence in the Mesozoic (Figure 5), extending the fossil record of the group back in time by at least ∼10 Ma, and possibly more (up to ∼28 Ma, depending on the uncertainty surrounding the age of the Adamantina Formation). The results of the phylogenetic analysis presented here (Figure 4), which recovers *Boipeba* as the sister group to the Typhlopoidea, is consistent with molecular estimates for the origin of the group during the Cretaceous (Zheng and Wiens, 2016; Pyron and Wallach, 2014; Burbrink et al., 2020).

The presence of *Boipeba* in South America has implications for the biogeographic history of the Typhlopoidea, supporting a possible western Gondwana (South American) origin for the group (e.g., Pyron and Wallach, 2014), instead of an eastern Gondwana (India-Madagascar) origin followed by the breakup of Pangaea (contra ref. Vidal et al., 2010). This fossil finding together with the recent studies on molecular divergence age estimates (Pyron and Wallach, 2014; Zheng and Wiens, 2016) is most consistent with a Cretaceous (∼122 Ma) rather than the initially hypothesized Middle Jurassic age for the clade (∼150 Ma) proposed by Vidal et al. (2010).

The position of Boipeba on the typhlopoid lineage means that it can be used as a calibration (66–87.8Ma) for the minimum age of the typhlopoid-leptotyphlopid divergence, in molecular divergence studies.

*Boipeba tayasuensis* is estimated to have been slightly over 1 m in TL, a giant among scolecophidians, which are typically less than 30 cm (Hedges, 2008; Feldman et al., 2016) (Figures 4B and 4C). Its size is more comparable to that of typical alethinophidians (excluding boas and pythons), as well as of some early fossil stem snakes (Figure 4 and Data S2). This unusually large size provides insights into the early evolution of body size in blind snakes, suggesting that the ancestral scolecophidian was a sizable snake. When *Boipeba* is included in analyses, the estimated total length of 39 cm of the scolecophidian ancestor is considerably larger than the average TL of extant members of the clade; the same applies to the estimated total length of the ancestral typhlopoid (40 cm). This scenario is even more dramatic when considering the most recent common ancestor (MRCA) of *Boipeba* and Typhlopoidea, where the ancestral state reconstruction produced a TL estimate of 51 cm, nearly twice the length of the average living typhlopoid (see Results and Figure S9). Thus, the miniaturized body plan of modern scolecophidians represents a trait that evolved later in the group, rather than its ancestral condition. Furthermore, our ancestral state reconstruction suggests that miniaturization evolved independently in the three blind snake lineages (Anomalepididae, Leptotyphlopidae, and Typhlopoidea: Figure S9), as previously suggested by some molecular studies (e.g., Harrington and Reeder, 2017).

Living blind snakes that approach the size of *Boipeba* are extremely rare and include members of the typhlopoids such as the two closely related African species *Afrotyphlops schlegelii* and *Afrotyphlops mucruso*, which can achieve a TL of almost 1 m (List, 1966; Broadley and Wallach, 2009). However, in the Cretaceous such large size may have been more common, if not the norm, for early scolecophidians; the small size of post-Cretaceous forms might be due to the strong selective pressure imposed by the K/Pg extinction event, where smaller cryptic animals may have had greater chances of survival and subsequent diversification (Figure 4) (Longrich et al., 2012b; Klein, 2019). If true, then this has important implications for the debate on the origin of snakes, where miniaturized burrowers similar to blind snakes have been postulated to be ancestral to modern snakes, if not all snakes (e.g., Miralles et al., 2018). *Boipeba* suggests that the small body size of living blindsnakes does not characterize early blindsnakes, and cannot be extrapolated to early snakes in general.

Taken together, our findings provide a new perspective on the evolution of scolecophidians and early snakes. *Boipeba* provides evidence that blind snakes were already present and relatively large in the Mesozoic, and that the small size of living members of this group is likely due to subsequent miniaturization. Finally, the discovery of a scolecophidian in the Late Cretaceous of South America provides a crucial new calibration point for future molecular studies of divergence times within Serpentes.

### Limitations of the Study

Our phylogenetic analyses robustly united *Boipeba* with living blind snakes (scolecophidians), but did not robustly resolve its placement within Scolecophidia. This is likely due to the limited number of informative phylogenetic characters that could be scored from vertebral characters alone. The lack of absolute dating of the minimum age of the fossil locality hampers a more precise estimate for the age of this fossil, which impacts the estimate of the size of the ancestral scolecophidian. However, our analyses consider the most conservative minimum age (66 Ma), which means the fossil could be much older and thus closer in time to the ancestral blind snake. This means that the large size of *Boipeba* would exert a stronger influence on the estimated size of the ancestral blind snake. Thus, a tighter (older) constraint on the minimum age of *Boipeba* would potentially improve support for the body size patterns retrieved here.

### Resource Availability

#### Lead Contact

Further information and requests for resources and reagents should be directed to and will be fulfilled by the Lead Contact, Thiago Schineider Fachini (thiagoschineiderf@usp.br)

#### Materials Availability

The fossil is housed at the Museum of Palaeontology “Prof. Antônio Celso de Arruda Campos,” Monte Alto, São Paulo State, Brazil. All the comparative material including fossils and extant specimens used for this study are housed at public institutions and thus accessible to scientists, and a list with all relevant specimens, CT scan imagery (including original slice data), and scripts for all phylogenetic and comparative analyses can be found in the Transparent Methods section in the supplemental file.

#### Data and Code Availability

All the relevant data for this study such as the used scripts for the phylogenetic analyses together with the unprocessed datasets, the surface reconstruction file, the supplementary figures in full resolution, and the raw CT-Scan slices are freely available at Mendeley Data repository (https://doi.org/10.17632/4dh8fj54f6.1). Original data have been deposited to Mendeley Data: [https://doi.org/10.17632/4dh8fj54f6.1].

## Methods

All methods can be found in the accompanying Transparent Methods supplemental file.

## Supplemental Information

Document S1. Transparent Methods and Figures S1–S10

Data S1. Body Length Data Used for the Estimate of the Size of Boipeba, Related to Figure 4

Data S2. Data of Snake Body Length Histogram Plots, Related to Figure 4
