# Cretaceous blind snake from Brazil fills major gap in snake evolution

**DOI:** 10.1016/j.isci.2021.102180

**Published:** 2021-02-24

**Authors:** Thiago Schineider Fachini, Silvio Onary, Alessandro Palci, Michael S.Y. Lee, Mario Bronzati, Annie Schmaltz Hsiou

## Main text

(iScience *23*, 101834-1–101834-13; December 18, 2020)

In the originally published version of this article, the Zoobank registration number for the described snake species has been omitted from the Data and Code Availability section. This has now been added in the article online. The authors apologize for any confusion this error may have caused.

