# Supplementary material for: Cretaceous Blind Snake from Brazil Fills Major Gap in Snake Evolution
Source: iScience. 2020 Nov 20;23(12):101834. doi: 10.1016/j.isci.2020.101834 (PMC7718481; doi:10.1016/j.isci.2020.101834)
Supplement: Document S1. Transparent Methods and Figures S1–S10 [file mmc1.pdf]

iScience, Volume *23*

Supplemental Information

Cretaceous Blind Snake from Brazil Fills

Major Gap in Snake Evolution

Thiago Schineider Fachini, Silvio Onary, Alessandro Palci, Michael S.Y. Lee, Mario Bronzati, and Annie Schmaltz Hsiou

Supplemental Information

Transparent Methods

Materials

The morphological comparative analyses were conducted after first-hand examination of specimens supplemented by relevant publications in the literature. Extant and fossil snakes, as well the accessed literature used for the description and comparative sections include "parviraptorids" (Caldwell et al., 2015); *Dinilysia patagonica* (MACN-RN unnumbered specimen) (Caldwell and Albino, 2003); *Najash* spp. (Garberoglio et al., 2019b; Zaher et al., 2009; Garberoglio et al., 2019a); "Madtsoiids" such as *Wonambi naracoortensis* (SAMA P16168), Pachyophiidae (Rage and Escuillié, 2000), *Coniophis precedens* (UALVP unnumbered specimen) (Longrich et al., 2012; Rage, 1984); *Boipeba tayasuensis* MPMA 16-0008-08; *Afrotyphlops punctatus* USNM 320704; *Afrotyphlops angolensis* AMNH 116633; *Typhlops jamaicensis* AMNH R160154; *Anilios (=Ramphotyphlops) pinguis* SAMA R924; *Ramphotyphlops polygrammicus* SAMA R3564; *Ramphotyphlops proximus* SAMA R915; *Ramphotyphlops ligatus* SAMA R2820; *Anilius scytale* AMNH R55613, AMNH R155256, MCZ 19537; *Cylindrophis ruffus* MNHN 1869 771, SAMA R36779; *Simalia (=Morelia) amethistina* SAMA R2605.

Institutional abbreviations

AMNH American Museum of Natural History, New York, USA.

MACN-RN Museo Argentino de Ciencias Naturales 'Bernardino Rivadavia', Buenos Aires, Argentina.

MPMA Museu de Paleontologia Prof. Antônio de Arruda Campos, São Paulo, Brazil.

MNHN Muséum national d'Histoire Naturelle, Paris, France.

MCZ Museum of Comparative Zoology, Cambridge, Massachusetts, USA.

USNM National Museum of Natural History, Washington, DC, USA.

SAMA R-REPTILES OR P-PALEONTOLOGY South Australian Museum of Adelaide, South Australia, Australia.

UALVP University of Alberta Laboratory for Vertebrate Paleontology, Edmonton, Alberta, Canada.

Methods

Anatomical nomenclature

Vertebral anatomical nomenclature for the description and comparisons follows Rage (1984); Auffenberg (1963); and Hoffstetter and Gasc (1969).

Phylogenetic analysis

To investigate the phylogenetic affinities of *Boipeba tayasuensis*, we added it to the data matrix derived from a recent study on snake evolution (Garberoglio et al., 2019a), along with four additional vertebral characters from Goméz et al. (2019) and one new (this study), resulting in dataset I - Morphology. A second dataset was built combining this morphological data matrix with the molecular data derived from a broad scale genomic study of squamates (Tonini et al., 2016), resulting in dataset II - Morphology plus DNA (See Supplementary data 3). These character-by-taxon matrices were assembled in the software Mesquite (Maddison and Maddison, 2019). Datasets I and II were each analysed using either all 37 taxa, or the 33 most complete taxa (taxa with >90% missing data were excluded). Analyses used both maximum parsimony and Bayesian Inference, resulting in eight analyses (see supplementary figures 3,4). The maximum parsimony analyses were performed using PAUP (Swofford, 2003), with *Varanus* as the outgroup, and among the 253 equally weighted morphological characters, 21 were multistate morphoclines and thus treated as ordered (data matrix in

transparent methods). The most parsimonious trees (MPTs) were inferred using Heuristic Search with 100 random addition sequence (RAS) replicates, and a strict consensus was obtained. Support values were calculated via TNT using the partitioned bootstrap (Siddal, 2010), with the morphology and DNA data resampled separately (supplementary figure 5). Bayesian inference analyses were performed in the software Mr. Bayes v 3.2.6 (Ronquist et al., 2012) employing the Mkv model (Lewis, 2001) with gamma rate variation for the morphological partition, while (for Dataset II) the DNA partitions and models were selected using PartitionFinder 2 (Lanfear et al., 2017). The analyses using dataset I (morphology only) were performed with four independent runs of 40 million generations, each run using 4 chains (1 heated and 3 cold), with the chains being sampled every 2000 generations, heating set to 0.06, and burn-in fraction of 25%. The analysis using dataset II (molecular + morphology) used similar settings, but with 12 chains per run (1 heated and 11 cold), temperature parameter set to 0.07, and burn-in fraction of 40%. The convergence in the posterior distribution was confirmed with high effective sample size (ESS > 200) for each parameter, potential scale reduction factors (PSRF) approaching 1, and the low standard deviation of split (clade) frequencies across runs (ASDSF < 0.01). Our analyses were not dated, but for visualization purposes the phylogenies were time-scaled in Fig. 4 using the stratigraphic ages of occurrence for the fossils and estimated divergence time dates derived from a recent phylogenomic study of squamates (Zheng and Wiens, 2016). Four additional phylogenetic analyses employing the same parameters were also conducted with the inclusion of the enigmatic taxon *Tetrapodophis amplectus* in the snake ingroup (supplementary figs. 6-8); the position of *Boipeba* within scolecophidians remains supported, but we are cautious not to overinterpret the topological results for *Tetrapodophis* due to its disputed ophidian affinities (Caldwell et al., 2016; Paparella et al. 2018; Caldwell, 2019). All the used scripts

and data matrix for the phylogenetic analyses can be freely downloaded at Mendeley Data Repository (doi: 10.17632/4dh8fj54f6.1).

Size estimate, snake body length plots and ancestral state reconstruction

The total length (TL) of *Boipeba tayasuensis* was estimated using the ratio between the average centrum length (CL) and total length including tail (TL) in four different taxa of extant typhlopoids, each represented by 1 specimen. We subdivided the trunk (precloacal) region of each specimen into five intervals of equal length, and sampled a trunk vertebra from each boundary between intervals (i.e. 4 trunk vertebrae per specimen). We measured the centrum length (along the ventral margin) of these 4 trunk vertebrae using a Zeiss microscope micrometer, and then calculated the average CL. We then calculated the ratio (R) between the TL of each of the four sampled specimens and its average trunk CL. These ratios produced four distinct TL estimates for *Boipeba* based on its CL (CL_b) (i.e. estimated TL of *Boipeba* = R x CL_b). We took the average of the four estimates as our conservative TL estimate of *Boipeba* (see supplementary data 1).

In order to compare the estimated body size of *Boipeba* with that of other extinct and extant snakes, we plotted histograms of the distribution of the TL of all living species of each terminal clade of snakes used in our snake phylogeny and the TL of important fossil snakes obtained from the literature (Fig. 4; “parviraptorids” were excluded due to the fragmentary nature of the fossils). The size data for extant taxa were obtained from a broad scale study of body size in lepidosaurs (Feldman et al., 2016), and plotted on a log scale using using R (R Core Team R, 2013); for details see supplementary data 2).

Ancestral state reconstructions for scolecophidian body size were performed using parsimony/likelihood methods. To infer the ancestral size of the MRCA of Scolecophidia, we took the tree from a dated, well-sampled squamate phylogeny (Zheng and Wiens, 2016),

retaining only the 98 blindsnake species for which TL size data was available (Feldman et al., 2016) and pruning other taxa (nexus file available at Mendeley Data Repository [doi: 10.17632/4dh8fj54f6.1]) . TL was scored (on a log scale) for all these species, and ancestral states for all nodes were estimated via Mesquite (Maddison and Maddison, 2019) using squared-changed parsimony accounting for branch lengths, which is identical to the maximum likelihood estimates under Brownian motion (Maddison, 1991). In order to observe the impact of the fossil when estimating the TL of the common ancestor of all blindsnakes (see supplementary figure 9), the first analysis was conducted exclusively using the 98 extant species, then a second analysis was performed adding *Boipeba* as sister group to Typhlopoidea (mid-way along the typhlopoid stem, and with tip age corresponding to a midpoint estimate of the stratigraphic age of the Adamantina Formation; i.e. 76.85 Ma (87.78-66Ma).

CT scanning and image segmentation

High-resolution microCT scanning of *Boipeba tayasuensis* was conducted using a GE Phoenix v|tome|x S240 scanner at the Centro para Documentação da Biodiversidade, Departamento de Biologia (Universidade de São Paulo, Ribeirão Preto, Brazil). The virtual preparation and segmentation procedure were conducted in the software AVIZO lite v. 9.0, initially employing the threshold tool to remove the rock matrix, followed by manual slice-by-slice segmentation (in the three-axis view) using the brush and lasso tools. The segmented vertebra was then rendered as a surface file (.stl) for three-dimensional visualization. The raw CT-scan files together with the.stl file are available at Mendeley Data Repository (doi: 10.17632/4dh8fj54f6.1).

Systematic palaeontology of indeterminate ophidian material

Three articulated ophidian vertebrae were discovered in close proximity to the holotype of *Boipeba tayasuensis*. Despite the close association, a detailed comparison of the type material of *B. tayasuensis* with the other vertebrae does not support their assignment to the same taxon. (see below).

Systematic Palaeontology

Squamata Oppel, 1811

Ophidia Brongniart, 1800

Genus and species indet.

(Figure S10)

Material. Sequence of three fragmentary vertebrae (unregistered specimen).

Locality and horizon. Same from the type locality of *Boipeba tayasuensis*.

Description. The material comprises a series of three poorly preserved articulated ophidian vertebrae. The vertebrae can be confidently assigned to Ophidia due to the presence of well-developed zygosphenes and zygantra, and synapophyses subdivided into ventral and dorsal articulatory facets. The general morphology of the vertebrae is quite distinct when compared to *Boipeba* in having: higher neural spines; a concave anterior margin of the zygosphene roof; less depressed neural arch; trefoil-shaped cross section of the neural canal; the absence of prezygapophyseal processes; and a strong differentiation between ventral and dorsal articular facets of the synapophyses, with a convex diapophyseal articular facet and a subtriangular parapophyseal articular facet. The latter is ventrally oriented, and extends below the ventral margin of the cotyle. The synapophysis morphology is sharply distinct from the condition in *Boipeba*, which possesses a confluent (i.e. not divided) synapophysis. Due to poor preservation

and lack of diagnostic features, the taxonomic assignment of this material is hampered. Some features such as the absence of prezygapophyseal articular processes and the slightly concave anterior edge of the zygosphene (heart-shaped morphology) are also present but not exclusive to “madtsoiids”. The presence of distinct articular facets of the synapophyses is a feature widely distributed among extinct and extant snakes (with exception of *Boipeba* and scolecophidians). Given the morphological differences between *Boipeba* and the articulated series together with the lack of diagnostic morphological features, here we retain the conservative approach of identifying the material as indeterminate ophidian. The likely presence of a distinct indeterminate ophidian taxon co-occurring with *Boipeba* reinforces the underestimated snake fossil diversity of the Cretaceous of the Bauru Basin, which, aside from *Boipeba*, currently includes only two other non-described snake taxa (Onary et al., 2017). Only additional findings will help elucidate the taxonomic affinities of this fragmentary fossil snake.

About the raw data files

All the relevant data for this study such as the used scripts for the phylogenetic analyses together with the unprocessed datasets, the surface reconstruction file, the supplementary figures in full resolution and the raw CT-Scan slices are freely available at Mendeley Data repository (doi: 10.17632/4dh8fj54f6.1).

Character list used for the phylogenetic analyses

For the phylogenetics analyses we used the character list derived from Garberoglio et al., (2019b), along with four additional vertebral characters from Goméz et al., 2019 (249, 250, 252, 253) and one new (251). The following 21 characters were multistate morphoclines and thus treated as ordered: 13, 25, 44, 46, 56, 62, 78, 94, 108, 114, 117, 119, 128, 140, 167, 207, 225, 229, 234, 250, 253. Numbering of characters 1-248 follows Garberoglio et al., (2019a)

DENTITION

1. Maxillary and dentary teeth: relatively short conical, upright (0); robust, recurved (1); elongate needle-shaped, distinctly recurved (2).
2. Premaxillary dentition: present (0); absent (1).
3. Alveoli and base of teeth: not expanded transversely (0); wider transversely than anteroposteriorly (1).
4. Pterygoid teeth: absent (0); present (1).

SKULL

5. Premaxilla: broadly articulated with maxilla (0); loosely contacting maxilla (1).
6. Transverse processes of premaxilla: curved backwards (0); extending straight laterally or anterolaterally (1).
7. Nasal process of premaxilla: elongate, approaching or contacting frontals (0); short, divide nasals only at anterior margin or not at all (1).
8. Dorsal (horizontal) lamina of nasal: relatively broad anteriorly, with narrow gap between lateral margin and vertical flange of septomaxilla (0); dorsal lamina of nasal distinctly tapering anteriorly, leaving wide gap between lateral margin and vertical flange of septomaxilla (1).
9. Medial flanges of nasal, articulation with median frontal pillars: present (0); absent (1)
10. Anterior margin of nasals: restricted to posteromedial margins of nares (0); extend anteriorly toward tip of rostrum (1).
11. Lateral flanges of nasals: articulate with anterior margin of frontals (0); separated from frontals (1).
12. Posterolateral margin of nasal: contacts anteromedian margin of prefrontal (0); elements in contact along most of their length (1); contact between elements with interfingering of nasal and prefrontal margins (2); nasals do not contact prefrontals (3).
13. Septomaxilla posterior dorsal process of lateral vertical flange: absent (0); short (1); long (2).
14. Septomaxilla articulation with median frontal pillars: absent (0); present (1).
15. Ventral portion of posterior edge of lateral flange of septomaxilla and opening of Jacobsen’sorgan: located at level of posterior edge or behind (0); distinctly in front (1).
16. Vomeronasal cupola: fenestrated medially (0); closed medially by a sutural contact of septomaxilla and vomer (1).
17. Septomaxilla: forms lateral margin of opening of Jacobson’s organ (0); vomer extends into posterior part of lateral margin, restricting septomaxilla to anterolateral part of lateral margin of opening of Jacobson’s organ (1).
18. Vomeronasal nerve: does not pierce vomer (0); exits vomer through single large foramen (1); through cluster of small foramina (2).
19. Posterior ventral (horizontal) lamina of vomer: long, parallel edged (0); short, tapering to pointed tip (1).
20. Posterior dorsal (vertical) lamina of vomer: well developed (0); reduced or absent (1).

21. Prefrontal: articulates with frontal laterally (0); anterolaterally (1).
22. Lateral margin of prefrontal: slanting anteroventrally (0); positioned vertically (1).
23. Lacrimal foramen on prefrontal: not completely enclosed (0); enclosed by prefrontal (1); prefrontal lacking foramen (2).
24. Lateral foot process of prefrontal: absent (0); contacts maxilla only (1); maxilla and palatine (2); palatine only (3).
25. Medial foot process of prefrontal: absent (0); present, low (1); present, high (2).
26. Anterior/lateral flange of prefrontal covering nasal gland and roofing auditus conchae: absent (0); present (1).
27.Ventral margin of lateral surface of prefrontal: articulates with dorsal surface of maxilla (0); retains only posterior contact (1).
28. Dorsal lamina of prefrontal: contacts or forms overlapping contact with nasal posteromedially (0); remains separate from nasal (1).
29. Medial frontal pillars: absent (0); present (1).
30. Transverse horizontal shelf of frontal: developed and broadly overlapped by nasals (0); poorly developed and never broadly overlapped by nasals (1); absent (2).
31. Lacrimal: present (0); absent (1).
32. Postfrontal: present (0); absent (1).
33. Jugal: present (0); fused or absent (1).
34.Jugal, ventral tip: Contact or approaches prefrontal (or lacrimal), forming or contributing to ventral margin of orbit (0); contacts or closely approaches ectopterygoid/maxilla, forming almost complete posterior margin of orbit (1); remains separated by wide gap from ectopterygoid (2).
35.Jugal, dorsal head: contacts postorbital (0); contacts parietal (1); fuses or articulates with only the posterodorsal surface of postfrontal (2); lack of dorsal contact (3).
36. Parietal: without lateral wings meeting postorbital bones (0); with lateral wings meeting postorbital bones (1).
37. Distinct lateral ridge of parietal: extending posteriorly from anterior lateral wing up to prootic: absent (0); present (1).
38. Frontoparietal suture: relatively straight (0); frontoparietal suture U-shaped (1).
39. Optic foramen, posterior margin: posteriorly located, straight parietal margin (0), posteriorly located, concave parietal margin (1); anteriorly located, posterior border within frontal (2).
40. Lateral margins of braincase open anterior to prootic (0); descending lateral processes of parietal enclose braincase (1).
41. Supratemporal processes of parietal: distinctly developed (0); not distinctly developed (1).
42. Parietal enters anterior aspect of base of basipterygoid process: absent (0); present (1).
43. Contact between parietal and supraoccipital: V-shaped with apex pointing anteriorly (0); straight transverse line (1); V-shaped with apex pointing posteriorly (2).
44. Ascending process of maxilla: tall, extending to dorsal margin of prefrontal (0); short (1); absent (2).
45. Small horizontal shelf on medial surface of anterior end of maxilla: present (0); absent (1).
46. Posterior end of maxilla: does not project beyond posterior margin of orbit (0); projects moderately beyond posterior margin of orbit (1); projects distinctly beyond posterior margin of orbit, with broad flat surface (2).
47. Medial (palatine) process of maxilla: located in front of orbit (0); located below orbit (1).
48. Medial (palatine) process of maxilla: pierced (0); not pierced (1).
49. Anterior end of supratemporal: located behind or above posterior border of trigeminal

foramen (0); anterior to posterior border of trigeminal foramen (1).

50. Supratemporal facet on opisthotic-exoccipital: flat (0); sculptured and delineated with projecting posterior rim that overhangs exoccipital (1).

51. Free-ending posterior process of supratemporal: absent (0); present (1).

52. Supratemporal: present (0); absent (1).

53. Anterior dentigerous process of palatine: absent (0); present (1).

54. Medial (choanal) process of palatine: forms extensive concave surface dorsal to ductus nasopharyngeus (0); narrows abruptly to form curved finger-like process (1); forms short horizontal lamina that does not reach vomer (2).

55. Choanal process of palatine: without expanded anterior flange articulating with vomer (0); with anterior flange (1).

56. Pterygoid contacts palatine: complex and finger-like articulations (0); tongue-in-groove joint (1); reduced to flap-overlap (2).

57. Palatine contact with ectopterygoid: present (0); absent (1).

58. Dentigerous process of palatine contact with vomer and/or septomaxilla posterolateral to opening for Jacobson's organ: present (0); absent (1).

59. Maxillary process of palatine: anterior to posterior end of palatine (0); at posterior end of palatine (1).

60. Lateral (maxillary) process of palatine and maxilla: in well-defined articulation (0); loosely overlapping medial (palatine) process of maxilla, or absent (1).

61. Maxillary branch of trigeminal nerve: pierces lateral (maxillary) process of palatine (0); passes dorsally between palatine and prefrontal (1).

62. Vomerine (choanal) process of palatine: articulates broadly with posterior end of vomer (0); meets vomer in well-defined articular facet (1); touches or abuts vomer without articulation or remains separated from vomer (2).

63. Internal articulation of palatine with pterygoid: short (0); long (1).

64. Pterygoid tooth row: anterior to basipterygoid joint (0); tooth row reaches or passes level of basipterygoid joint (1).

65. Quadrate ramus of pterygoid: robust, rounded or triangular in cross-section, but without groove (0): blade-like and with distinct longitudinal groove for protractor pterygoidei (1).

66. Transverse (lateral) process of pterygoid: forms distinct, well-defined lateral projection (0); gently curved lateral expansion of pterygoid, or absent (1).

67. Lateral edge of ectopterygoid: straight (0); angulated at contact with maxilla (1).

68. Anterior end of ectopterygoid: restricted to posteromedial edge of maxilla (0); invades dorsal surface of maxilla (1).

69. Pterygoid attached to basicranium: by strong ligaments at palatobasal articulation (0); pterygoid free from basicranium in dried skulls (1).

70. Quadrate: slender (0); broad (1).

71. Quadrate: slanted clearly anteriorly, posterior tip of pterygoid dislocated anteriorly from mandibular condyle of quadrate (0); positioned slight anteriorly or vertically (cephalic condyle positioned behind or at same level of mandibular condyle) (1); slanted posteriorly (cephalic condyle positioned in front of mandibular condyle) (2).

72. Cephalic condyle of quadrate: elaborated into posteriorly projecting suprastapedial process (0); suprastapedial process absent or vestigial (1).

73. Stapedial footplate: broad and massive (0); narrow and thin (1).

74. Stylohyal: not fused to quadrate (0); fuses to posterior tip of suprastapedial process (1); fusesto ventral aspect of reduced suprastapedial process (2); stylohyal fuses to quadrate shaft (3).

75. Stapedial shaft: straight (0); angulated (1).

76. Stapedial shaft: slender and longer than diameter of stapedial foot-plate (0); thick, and

equal to, or shorter than diameter of stapedial footplate (1).

77. Paroccipital process of otooccipital: well developed and laterally projected (0); reduced to short projection or absent (1).

78. Juxtastapedial space defined by a crista prootica, crista tuberalis and crista interfenestralis: absent (0); present, but not completely enclosed ("incipient"crista circumfenestralis) (1); present and enclosed (i.e., fully developed crista circumfenestralis) (2).

79. Stapedial footplate: mostly exposed laterally (0); Prootic and otoocipital converges upon stapedial footplate (1).

80. Crista interfenestralis: does not form individualized component around the juxtastapedial space (0); does form individualized component around juxtastapedial space (1).

81. Jugular foramen: exposed in lateral view by crista tuberalis (0); concealed in lateral view by crista tuberalis (1).

82. Otooccipitals: do not contact each other dorsally (0); contact each other dorsally (1).

83. Basioccipital posterolateral processes: short and narrow, do not extend toward posterior margin of occipital condyle (0); wider than condyle and long, combine with crista tuberalis to extend to approximate posterior margin of occipital condyle (1).

84. Supraoccipital contact with prootic: narrow (0); broad (1).

85. Prootic exclusion of parietal from trigeminal foramen: absent (0); present (1).

86. Laterosphenoid: absent (0): present (1).

87. Prootic ledge underlap of posterior trigeminal foramen: absent (0); present (1).

88. Prootic: exposed in dorsal view medial to supratemporal or to supratemporal process of parietal (0); fully concealed by supratemporal or parietal in dorsal view (1).

89. Exit hyomandibular branch of facial nerve inside opening for mandibular branch of trigeminal nerve: absent (0); present (1).

90. Vidian canal: does not open intracranially (0); open intracranially (1).

91. Anterior opening of Vidian canal: single (0); divided (1).

92. Sella turcica: bordered posteriorly by well-developed dorsum sellae (0); dorsum sellae low (1); dorsum sellae not developed, sella turcica with shallow posterior margin (2).

93. ‘Lateral wings of basisphenoid’: absent (0); present (1).

94. Ventral surface of basisphenoid: smooth (0); with weakly developed sagittal crest from which protractor pterygoidei originates (1); with strongly projecting sagittal crest (2).

95. Basioccipital: contributes to ventral margin of foramen magnum (0); basioccipital excluded by medial contact of otooccipitals (1).

96. Basisphenoid-basioccipital suture: smooth (0); transversely crested (1).

97. Basipterygoid (= basitrabecular) processes: present (0); absent (1).

98. Crista trabeculares: short and or indistinct (0); elongate and distinct in lateral view (1).

99. Cultriform process of parabasisphenoid: does not extend anteriorly to approach posteriormargin of choanae (0); approaches posterior margin of vomer (1).

100. Parabasisphenoidal rostrum behind optic foramen: narrow (0); broad (1).

101. Parabasisphenoid rostroventral surface: flat or broadly convex (0); concave (1).

102. Basioccipital meets parabasisphenoid: suture located at level of fenestra ovalis (0); located at or behind trigeminal foramen (1); basioccipital and parabasisphenoid fused (2).

103. Parasphenoid rostrum interchoanal process: absent (0); broad (1); narrow (2).

MANDIBLE

104. Anteromedial margin of dentaries: symphyseal articular facet (0); no symphyseal facet (1).

105. Posterior dentigerous process of dentary: absent (0); present, short (1); present, long (2).

106. Medial margin of adductor fossa: relatively low and smoothly rounded (0); forms

distinct dorsally projecting crest (1).
107. Mental foramina on lateral surface of dentary: two or more (0); one (1).
108. Coronoid process of coronoid bone: high, tapering distally (0); high, with rectangular shape (1); low, not exceeding significantly coronoid process of compound bone (2).
109. Coronoid bone: present (0); absent (1).
110. Posteroventral process of coronoid: present (0); absent (1).
111. Coronoid process on lower jaw: formed by coronoid bone only (0); or by coronoid and compound bone (1); or by compound bone only (i.e. coronoid absent) (2).
112. Posdentary elements: presence of separate elements (0); fusion of surangular /articular intocompound bone (1).

VERTEBRAE
113. Chevrons: present (0); absent (1).
114. Hemapophyses: absent (0); present, short (1); present, long (2).
115. Hypapophyses: restricted to anterior-most precloacal vertebrae (0); present throughout precloacal skeleton (1).
116. Para-diapophysis: confluent (0); separated into dorsal and ventral facet (1).
117. Prezygapophyseal accessory processes: absent (0); present, short (1); present, long
118. Subcentral paralymphatic fossae on posterior precloacal vertebrae: absent (0); present (1).
119. Subcentral foramina: absent (0); present, consistently small (1); present, of variable size (2).
120. Well-developed, consistently distributed paracotylar foramina: absent (0); present (1).
121. Ventral margin of centra: smooth (0); median prominence from cotyle to condyle (1).
122. Axis intercentrum: not fused to anterior region of axis centrum (0); fused (1).
123. Neural spine height: well-developed process (0); low ridge or absent (1).
124. Posterior margin of neural arch: shallowly concave in dorsal view (0); with deep V-shaped embayment in dorsal view (1).
125. Cotyle shape of precloacal vertebrae: oval (0); circular (1).
126. Parazygantral foramen: absent (0); present (1).
127. Lymphapophyses: absent (0); present (1).
128. Lymphapophyses: three or fewer (0); three lymphapophyses and one forked rib (1); more than three lymphapophyses and one forked rib (2).
129. Sacral vertebrae: present (0); absent (1).
130. Position of synapophyses in relation to lateral edge of prezygapophyses: at same level or slightly more projected laterally (0); clearly medial to edge of prezygapophyses (1).
131. Pachyostotic vertebrae: absent (0); present (1).
132. Precloacal vertebrae number: fewer than 100 (0); more than 100 (1).
133. Caudal vertebrae number: greater than 50% of precloacal number (0); approximately 10% or less than precloacal number (1).
134. Tuber costae absent from ribs (0), tuber costae present (1).

HINDLIMBS
135. Pectoral girdle and forelimbs: present (0); absent (1).
136. Tibia, fibula, and hind foot: present (0); absent (1).
137. Trochanter externus: present (0); absent (1).
138. Pelvis: external to sacral-cloacal ribs (0); internal to sacral-cloacal ribs (1).
139. Ilium and pubis length: ilium longer than pubis (0); ilium and pubis of same size (1); pubis much longer than ilium (2).
140. Pelvic elements: with strongly sutured contact (0); with weak (cartilaginous) contact (1);

fused together (2).

141. Pelvic elements: present (0); absent (1).

142. Medial vertical flanges of nasals: absent (0); present (1).

143. Preorbital ridge: dorsally exposed (0); overlapped by prefrontal (1).

144. Lateral foot process of prefrontal: articulates with lateral edge of maxilla via thin anteroposteriorly directed lamina (0); articulates with maxilla via large contact that runs from lateral to medial dorsal surface of maxilla (1).

145. Medial finger-like process of ectopterygoid articulating with medial surface of maxilla:present (0); absent (1).

146. Posterolateral corners of basisphenoid: strongly ventrolaterally projected (0); not projected (1).

147. Basioccipital: expanded laterally to form floor of recessusscalae tympani (0); excluded from floor of recessusscalae tympani by otooccipital (1).

148. Frontal subolfactory process: absent or present as simple horizontal lamina (0); present and closing tractus olfactorius medially (1).

149. Ectopterygoid contact with pterygoid: restricted to transverse (lateral) process of pterygoid (0); contact expanded significantly on dorsal surface of pterygoid body (1).

150. Maxillary process of palatine: main element bridging contact with maxilla and palatine inventral view (0); covered ventrally by expanded palatine process of maxilla (1).

151. Coronoid bone contributes to anterior margin of adductor fossa: present (0); absent (1).

152. Coronoid bone: sits mostly on dorsal and dorsomedial surfaces of compound bone, being exposed in both lateral and medial views of mandible (0); applied to medial surface of compound bone (1).

TEETH

153. Teeth, implantation: interdental ridges absent (0): interdental ridges present (1).

154. Teeth, replacement: replacement teeth lie vertically (0); lie horizontally in jaws (1).

155. Teeth, replacement: single replacement tooth per tooth position (0); two or more replacement teeth per tooth position (1).

156. Teeth, attachment: ankylosed to jaws (0); teeth loosely attached by connective tissue (1).

157. Teeth, size: crowns isodont or enlarged at middle of tooth row (0) crowns large anteriorly,and decrease in size posteriorly (1); anterior teeth conspicuously elongate, length of crown significantly exceeds height of dentary at midlength (2).

SKULL

158. Premaxilla: ascending process transversely expanded, partly roofing external nares (0); ascending process mediolaterally compressed, blade-like or spine-like (1).

159. Premaxilla: premaxilla medial to maxillae (0); located anterior to maxillae (1).

160. Prefrontal: prefrontal socket for dorsal peg of maxilla absent (0); present (1).

161. Prefrontal extends medially across frontal for more than 75% of width of frontal: absent (0); present (1).

162. Expanded naris: Weakly developed naris (0); strongly concave anterior margin of prefrontal bordering naris (1).

163. Frontal: nasal processes of frontal project between nasals (0); nasal processes absent (1).

164. Frontals: frontals taper anteriorly, distinct interorbital constriction (0); frontals broad anteriorly, interorbital region broad (1).

165. Frontal: subolfactory process abuts prefrontal in immobile articulation (0); subolfactory process articulates with prefrontal in mobile joint (1); subolfactory process with distinct lateral peg or process that clasped dorsally and ventrally by prefrontal (2).

166. Frontals and parietals: do not contact ventrally (0); descending wings of frontals and

parietals contact ventrally to enclose optic foramen (1).

167. Parietal, sagittal crest: absent (0); present posteriorly but not anteriorly, and extending for no more than 50% of parietal midline length (1); present anteriorly and posteriorly, and extending more than 50% of parietal midline length (2).

168. Parietal: narrow (0); inflated (1).

169. Parietal. Posteriorly broad parietal (0); posteriorly narrow parietal (1)

170. Skull, postorbital region relative length: short, less than half (0); elongate, half or more (1).

171. Supraoccipital region of skull: nuchal crests absent (0); present (1).

172. Supratemporal: supratemporal short, does not extend posterior to paroccipital process (0); elongate, extending well beyond paroccipital process (1).

173. Maxilla: palatine process short, weakly developed (0); palatine process long, strongly projecting medially (1).

174. Maxilla, premaxillary process: medial projection articulating with vomers present (0); premaxillary process does not contact vomers (1).

175. Maxilla, number of mental foramina: 5 or more (0); 4 or fewer (1).

176. Maxilla, supradental shelf development: extending full length of maxilla (0); reduced anterior to palatine process (1).

177. Maxilla, medial surface of facial process with distinct naso-lacrimal recess demarcated dorsally by anteroventrally trending ridge: present (0); absent (1).

178. Maxilla, medial surface of facial process with well-defined fossa for lateral recess of nasal capsule: present (0); reduced and present as small fossa on back of facial process (1); absent, fossa for lateral recess developed entirely on prefrontal (2).

179. Maxilla: extensive contact of dorsal margin of maxilla with nasal (0); nasal-maxilla contact lost (1).

180. Maxilla: maxilla overlaps prefrontal laterally in tight sutural connection (0); overlap reduced, mobile articulation (1).

181. Maxilla: palatine process of maxilla projects medially (0); palatine process of maxilla downturned (1).

182. Maxilla, superior alveolar foramen: positioned near middle of palatine process, opening posterodorsally (0); positioned near anterior margin of palatine process, opening medially (1).

183. Maxilla, accessory foramen posterior to palatine process: absent (0); present (1).

184. Maxilla, ectopterygoid process: absent (0); present (1).

185. Maxilla: 15 or more maxillary teeth (0); fewer than 15 maxillary teeth (1); maxilla without teeth (2).

186. Postfrontal: anterior and posterior processes clasping frontals and parietals (0); anterior and posterior processes present, but postfrontal abuts frontals and parietals (1); anterior and posterior processes absent (2).

187. Supratemporal: free caudal end of supratemporal projects posteroventrally (0); posteriorly or posterodorsally (1).

188. Quadrate, lateral conch: present (0); absent (1).

189. Quadrate, maximum length relative to proximal width: quadrate elongate, maximum length at least 125% of maximum width of quadrate head (0); quadrate short, length less than 125% of width of quadrate head (1).

190. Quadrate, proximal end plate-like: absent (0); present (1).

191. Palatine, dentition: teeth small relative to lateral teeth (0); enlarged, palatine teeth at least half diameter of posterior maxillary teeth (1); palatine lacking dentition (2).

192. Palatine, elongate lateral process projecting to lateral edge of orbit to articulate with caudal margin of prefrontal: absent (0); present (1).

193. Epipterygoid: present (0); absent (1).

194. Ectopterygoid: clasps pterygoid anteromedially (0); ectopterygoid overlaps pterygoid (1); ectopterygoid abuts pterygoid medially (2).
195. Vidian canals: posterior openings symmetrical (0); asymmetrical (1).
196. Exoccipital-opisthotic: horizontal, wing-like crista tuberalis absent (0); present (1).
197. Otooccipitals: do not project posteriorly to level of occipital condyle (0); project posteriorly to conceal occipital condyle in dorsal view (1).
198. Sclerotic ring: present (0); absent (1).

MANDIBLE

199. Dentary, enlarged mental foramen: absent (0); present (1).
200. Dentary, depth of Meckelian groove anteriorly: deep slot (0); shallow sulcus (1).
201. Dentary, angular process shape: posteroventral margin of dentary angular process weakly wrapped around underside of jaw (0); dentary angular process projects more nearly horizontally to wrap beneath jaw (1).
202. Dentary, angular process length relative to coronoid process: angular process distinctly shorter than coronoid process, former terminating well anterior to latter (0); subequal in length posteriorly (1).
203. Dentary, symphysis: weakly projecting medially (0); hooked inward and strongly projecting medially (1).
204. Dentary, ventral margin: unexpanded, medial margin of dentary straight in ventral view (0); expanded, medial margin crescentic in ventral view (1).
205. Dentary, coronoid process: wraps around surangular laterally and medially (0); broad and sits atop surangular (1).
206. Dentary, coronoid process with slot for medial tab of surangular: absent (0) or present (1).
207. Dentary, subdental shelf: present along entire tooth row (0); present only along posterior portion of tooth row (1); absent (2).
208. Surangular, dentary process with distinct triradiate cross-section: absent (0); present (1).
209. Surangular, adductor fossa: small or absent (0); extended caudally towards jaw articulation (1).
210. Surangular: ventrolateral surface of surangular bearing distinct crest for attachment of adductor muscles: absent (0); present (1).
211. Coronoid, lateral overlap of coronoid onto dentary: absent (0); present (1).
212. Splenial attachment to dentary above Meckel's canal: close throughout length (0); loose,with dorsal dentary suture confined to posterodorsal corner of splenial (1); contact with subdental shelf reduced to small spur of bone or contact lost entirely (2).
213. Splenial - angular articulation: splenial overlaps angular (0); splenial abuts against angular to form hinge joint (1).
214. Splenial, size: splenial elongate, extends more than half distance from angular to dentary symphysis (0); splenial short, extends less than half distance from angular to symphysis (1).
215. Splenial, anterior mylohyoid foramen: present (0); absent (1).
216. Angular, lateral exposure (with coronoid region pointing dorsally): angular broadly exposed laterally along length (0); angular narrowly exposed laterally (1).
217. Angular, length posteriorly relative to glenoid (quadrate articulation): relatively long,extends more than half distance from anterior end of angular to glenoid; (0) relatively short,half or less of distance to glenoid (1); very short, one third or less of distance to glenoid (2).
218. Surangular, enlarged anterior surangular foramen: absent (0); or present (1).
219. Coronoid eminence: well-developed (0); weakly developed or absent (1).

220. Glenoid, shape: quadrate cotyle shallow (0), anteroposteriorly concave and transversely arched, ‘saddle shaped’ (1).
221. Retroarticular process: retroarticular process elongate (0) or shortened (1).
222. Hypapophyses of anterior precloacals: short, about 50% length of centrum (0); long, subequal to or longer than centrum (1).
223. Vertebrae, ridge-like or bladelike ventral keels developed posterior to hypapophyses: absent (0); present (1).
224. Vertebrae, dorsolateral ridges of neural arch: absent (0); present (1).
225. Vertebrae, vertebral centrum: narrow in ventral view (0); broad and subtriangular in shape (1); broad and square (2).
226. Vertebrae, arterial grooves: absent in neural arch (0); present (1).
227. Vertebrae, posterior condyle: confluent with centrum ventrally (0); distinctly separated from centrum by groove/constriction between centrum and condyle (1).
228. Vertebrae: narrow, width across zygapophyses not significantly greater than distance from prezygapophyses to postzygapophyses (0); vertebrae wide, width across zygapophyses 150% of length or more (1).
229. Vertebrae, zygosphene anterior margin: deeply concave anterior edge (0); shallowly concave anterior edge (1); straight or slightly sinuous anterior edge (2).
230. Basioccipital, ventral surface: smooth (0); sagittal crest of parabasisphenoid extends into basiocciptal (1).
231. Vertebrae, zygosphene width, expressed as ratio of zygosphene width to cotyle width, in anterior view: wide, ratio close to or more than 1 (0); narrow, ratio significantly less than 1 (1).
232. Vertebrae, constriction index, expressed as neural arch minimal width to total width, measured at the level of the prezygapophyseal lateral edge: slight constriction, ratio equal to or more than 0.67 (0); marked constriction, ratio less than 0.67 (1).
233. Vertebrae, narrow and sharp haemal keel: absent (0); present (1).
234. Vertebrae, cotyle size, expressed as ratio of cotyle width to total width (measured as the interdiapophyseal width): big cotyle, ratio more than 0.5 (0); middle-sized cotyle, ratio between 0.5 and 0.3 (1); small cotyle, ratio less than 0.3 (2).
235. Vertebrae, small lateral ridge on precloacal vertebrae extending from the parapophyses, below lateral foramen: absent (0); present (1).
236. Supraoccipital, shape of dorsal exposure: broad and square (0); wider than longer, with broad edges (rectangular) (1); wider than long, with pointed medial edges (2); diamond-shaped (3); ‘M’-shaped (4); absent or fused (5).
237. Supraoccipital, size of dorsal exposure, expressed as ratio of supraoccipital length (measured at the midline) to parietal width (measured at the line delimited by the anterior borders of the prootic): big, ratio of 0.5 or more (0); small, ratio clearly less than 0.5 (1).
238. Vertebrae: unfused intercentra in precloacal vertebrae posterior to the axis, present (0); absent (1).
239. Jugal, distinct posterior process for quadratomaxillary ligament: present (0); absent (1).
240. Postorbital: present (0); absent (1)
241. Vertebrae, arqual ridges on middle precloacals: absent (0); present (1)
242. Pubis, obturator foramen: present (0); absent (1).
243. Ascending/facial process of maxilla, posterior notch on medial surface for prefrontal: present (0); absent (1).
244. Dentition, dentary teeth: present (0); absent (1).
245. Parietals: single (0); remain paired in adult skull (1)
246. Supraoccpitals: single (0); remain paired in adult skull (1)
247. Prootic: separated element (0); fused to braincase (1)

248. Ectopterygoid: present (0); highly reduced or absent (1).

249.Parapophysis ventral margin: high, placed dorsal to the ventral margin of cotyle (0); ventrally projected, level with or below ventral margin of cotyle (1).

250. Absolute size of neural spine, expressed as neural spine height (measured from dorsal edge of zygosphene) to total height of vertebra: high, >30% (0); moderate, between 15-30% (1); low, less than 15% (2).

251.Neural arch morphology flattened, dorsoventrally compressed (0); dorsoventrally expanded, vaulted (1).

252.Condyles of middle precloacal verterbrae, orientation: facing very dorsally, ventral edge (at most) of condyle surface exposed in ventral view (0); facing posteriorly, or posterodorsally, much of condyle surface exposed in ventral view (1).

253. Orientation of zygapophyses of middle precloacal vertebrae: steeply inclined medially, 30° or more from the horizontal (0); moderately inclined medially, between 15-30° from the horizontal (1); not inclined medially, <15° from horizontal (2).

Supplemental References

Auffenberg, W. (1963). The fossil snakes of Florida. University of Florida, Florida State Museum.

Caldwell, M. W., & Albino, A. (2003). Exceptionally preserved skeletons of the Cretaceous snake *Dinilysia patagonica* Woodward, 1901. J. Vertebr. Paleontol. *22,* 861-866.

Caldwell, M. W., Nydam, R. L., Palci, A., & Apesteguía, S. (2015). The oldest known snakes from the Middle Jurassic-Lower Cretaceous provide insights on snake evolution. Nat. Commun. 6, 1-11.

Caldwell, M. W., Reisz, R. R., Nydam, R. L., Palci, A. & Simões, T. R. (2016) *Tetrapodophis amplectus* (Crato Formation, Lower Cretaceous, Brazil) is not a snake. SVP Book of Abstracts. Society of Vertebrate Paleontology, Meeting program & Abstracts of the 76th Annual meeting, 108-108.

Caldwell, M.W. (2019). The origins of snakes: morphology and fossil record. Boca Raton: Taylor & Francis. 327 p. ISBN 9781482251340.

Feldman, A., Sabath, N., Pyron, R. A., Mayrose, I., & Meiri, S. (2016). Body sizes and diversification rates of lizards, snakes, amphisbaenians and the tuatara. Global Ecol. Biogeogr. *25,* 187-197.

Garberoglio, F.F., Apesteguía, S., Simões, T.R., Palci, A., Gómez, R.O., Nydam, R.L., Larsson, H.C., Lee, M.S.Y. & Caldwell, M.W. (2019a). New skulls and skeletons of the

Cretaceous legged snake *Najash*, and the evolution of the modern snake body plan. Sci. Adv. *5*, eaax5833.

Garberoglio, F. F., Gómez, R. O., Apesteguía, S., Caldwell, M. W., Sánchez, M. L., & Veiga, G. (2019b). A new specimen with skull and vertebrae of *Najash rionegrina* (Lepidosauria: Ophidia) from the early Late Cretaceous of Patagonia. J. Syst. Palaeontol. *17*, 1533-1550.

Gómez, R.O., Garberoglio, F.F. & Rougier, G.W. (2019). A new Late Cretaceous snake from Patagonia: Phylogeny and trends in body size evolution of madtsoiid snakes. CR. Palevol. *1136*, 1-11.

Hoffstetter, R., & Gasc, J. P. (1969). Vertebrae and ribs of modern reptiles. Biology of the Reptilia, 1(5), 201-310.

Lanfear, R., Frandsen, P. B., Wright, A. M., Senfeld, T., & Calcott, B. (2017). PartitionFinder 2: new methods for selecting partitioned models of evolution for molecular and morphological phylogenetic analyses. Mol. Phylogenetics Evol. *34*, 772-773.

Lewis, P. O. (2001). A likelihood approach to estimating phylogeny from discrete morphological character data. Syst. Biol. *50*, 913-925.

Longrich, N. R., Bhullar, B. A. S., & Gauthier, J. A. (2012). A transitional snake from the Late Cretaceous period of North America. *Nature, 488*(7410), 205-208.

Maddison, W. P. (1991). Squared-change parsimony reconstructions of ancestral states for continuous-valued characters on a phylogenetic tree. Syst. Biol. *40*, 304-314.

Maddison, W. P. and D.R. Maddison. (2019). Mesquite: a modular system for evolutionary analysis. Version 3.61 http://www.mesquiteproject.org.

Onary, S., Fachini, T.S., Hsiou, A.S. (2017). The snake fossil record from Brazil. J. Herpetol. *51*, 365-374.

Paparella, I., Palci, A., Nicosia, U., & Caldwell, M. W. (2018). A new fossil marine lizard with soft tissues from the Late Cretaceous of southern Italy. R. Soc. Open Sci. *5*, 172411.

R Core Team. R: A language and environment for statistical computing. R Foundation for Statistical Computing, Vienna, Austria. URL http://www.R-project.org/. (2013)

Rage, J.C. (1984). Part 11 Serpentes. In: Wellnhofer M, ed. Encyclopedia of paleoherpetology. Germany: Gustav Fischer Verlag, 1–79

Rage, J.-C. & Escuillié, F. (2000). Un nouveau serpent bipède du Cénomanien (Crétacé). Implications phylétiques. C. R. Acad. Sci. Paris (IIa) *330*, 513–520.

Ronquist, F., Teslenko, M., Van Der Mark, P., Ayres, D. L., Darling, A., Höhna, S., ... & Huelsenbeck, J. P. (2012). MrBayes 3.2: efficient Bayesian phylogenetic inference and model choice across a large model space. Syst. Biol. *61*, 539-542.

Siddall, M. E. (2010). Unringing a bell: metazoan phylogenomics and the partition bootstrap. Cladistics *26*, 444-452.

Swofford, D. L. (2003). PAUP: phylogenetic analysis using parsimony, version 4.0 b10.

Tonini, J. F. R., Beard, K. H., Ferreira, R. B., Jetz, W., & Pyron, R. A. (2016). Fully-sampled phylogenies of squamates reveal evolutionary patterns in threat status. Biol. Conserv. *204*, 23-31.

Zaher, H., Apesteguia, S., & Scanferla, C. A. (2009). The anatomy of the upper cretaceous snake *Najash rionegrina* Apesteguía & Zaher, 2006, and the evolution of limblessness in snakes. Zool. J. Linnean. Soc. *156*, 801-826.

Zheng, Y., & Wiens, J. J. (2016). Combining phylogenomic and supermatrix approaches, and a time-calibrated phylogeny for squamate reptiles (lizards and snakes) based on 52 genes and 4162 species. Mol. Phylogenetics Evol. 94, 537-547.

Supplementary figures

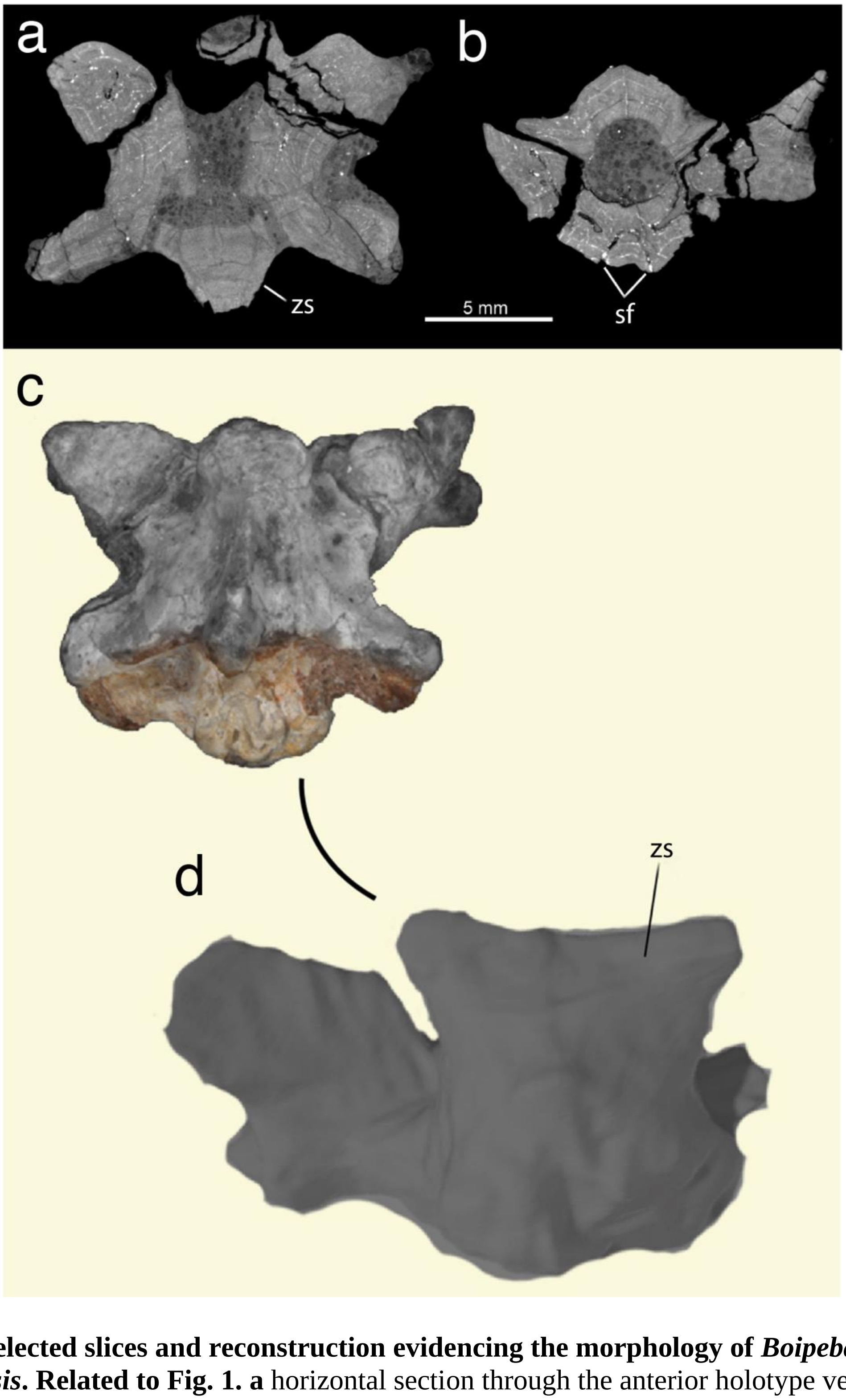

Fig. S1 Selected slices and reconstruction evidencing the morphology of *Boipeba tayasuensis*. Related to Fig. 1. a horizontal section through the anterior holotype vertebra showing the anterior edge of the zygosphene of the partial successive vertebra (anterior to the top). **b**. cross section through the holotype vertebra showing the presence of paired subcentral foramina. **c**. holotype evidencing the partial successive vertebra. **d**. three-dimensional

reconstruction of the partial successive vertebra showing the complete zygosphene roof morphology (anterior to the top). Sf., subentral foramina; ZS, zygosphene.

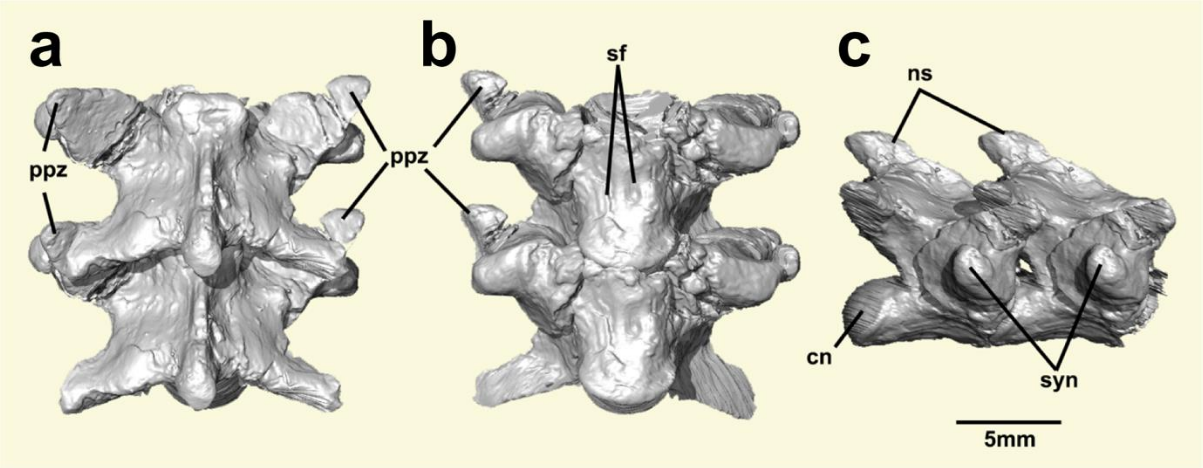

Fig. S2 Articulated sequence of digitally reconstructed vertebrae of *Boipeba tayasuensis*. Related to Fig. 2. a Dorsal view (anterior to the top). **b** Ventral view. **c** Right lateral view. This three-dimensional reconstruction is based on a digital replica articulated with the holotype. This image was generated with the free, open source software Blender v.2.79b. Note the distinct size of the elongated prezygapophyseal accessory processes of *Boipeba* protruding beyond the anterolateral margin of the prezygapophyseal facets. cn., condyle; ns., neural spine; ppz., prezygapophyseal accessory processes; sf., subcentral foramina; syn., synapophyses.

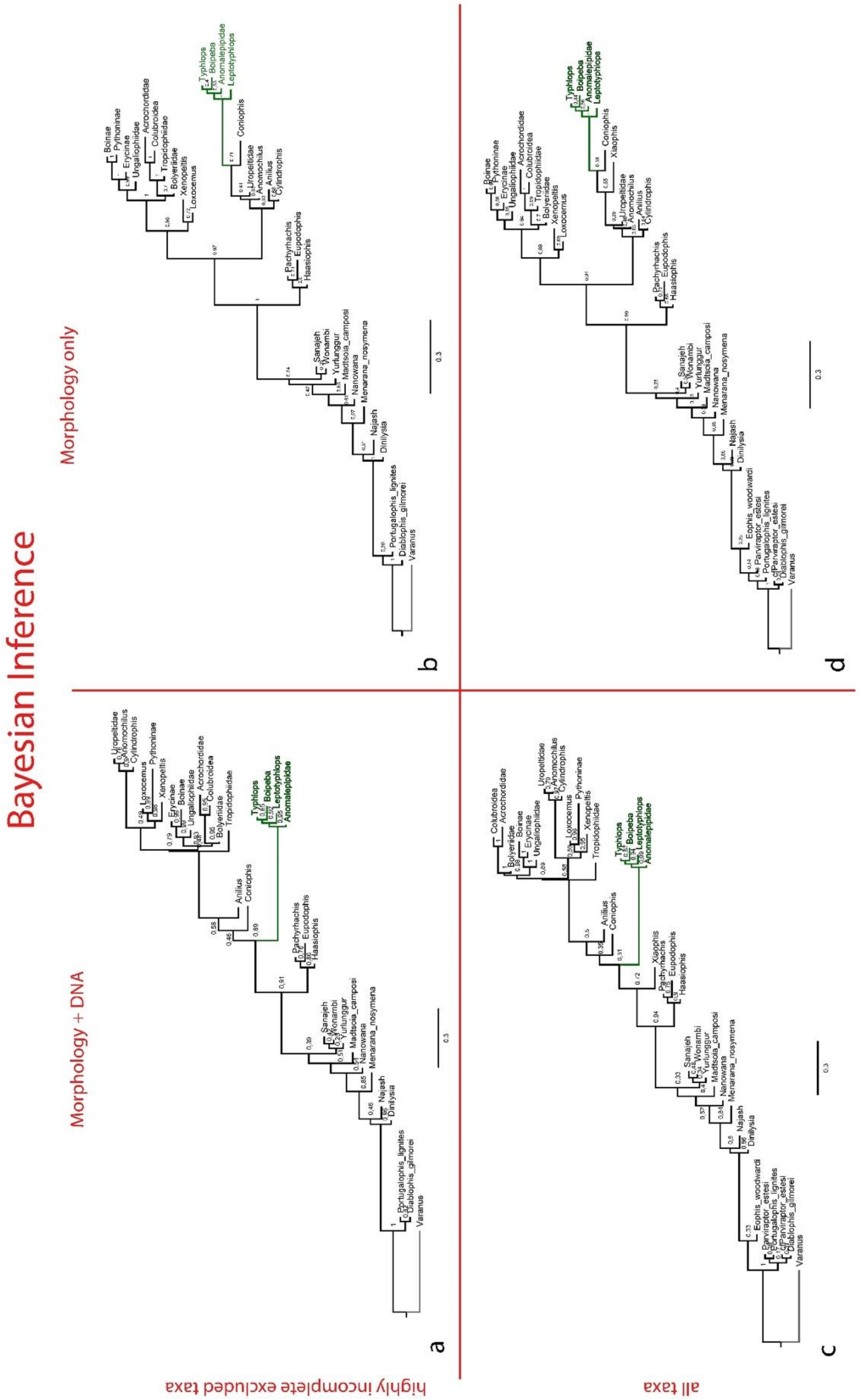

Fig. S3 Relationships of *Boipeba* and major snake lineages, based on Bayesian inference; majority-rule consensus trees from MrBayes. Related to Fig. 4. Numbers denote clade posterior probabilities. Scolecophidian (blindsnake) taxa in green. **a**. Morphology and DNA, with highly incomplete taxa excluded; **b**. Morphology only, with

highly incomplete taxa excluded; **c**. Morphology and DNA (all taxa); **d**. Morphology only (all taxa).

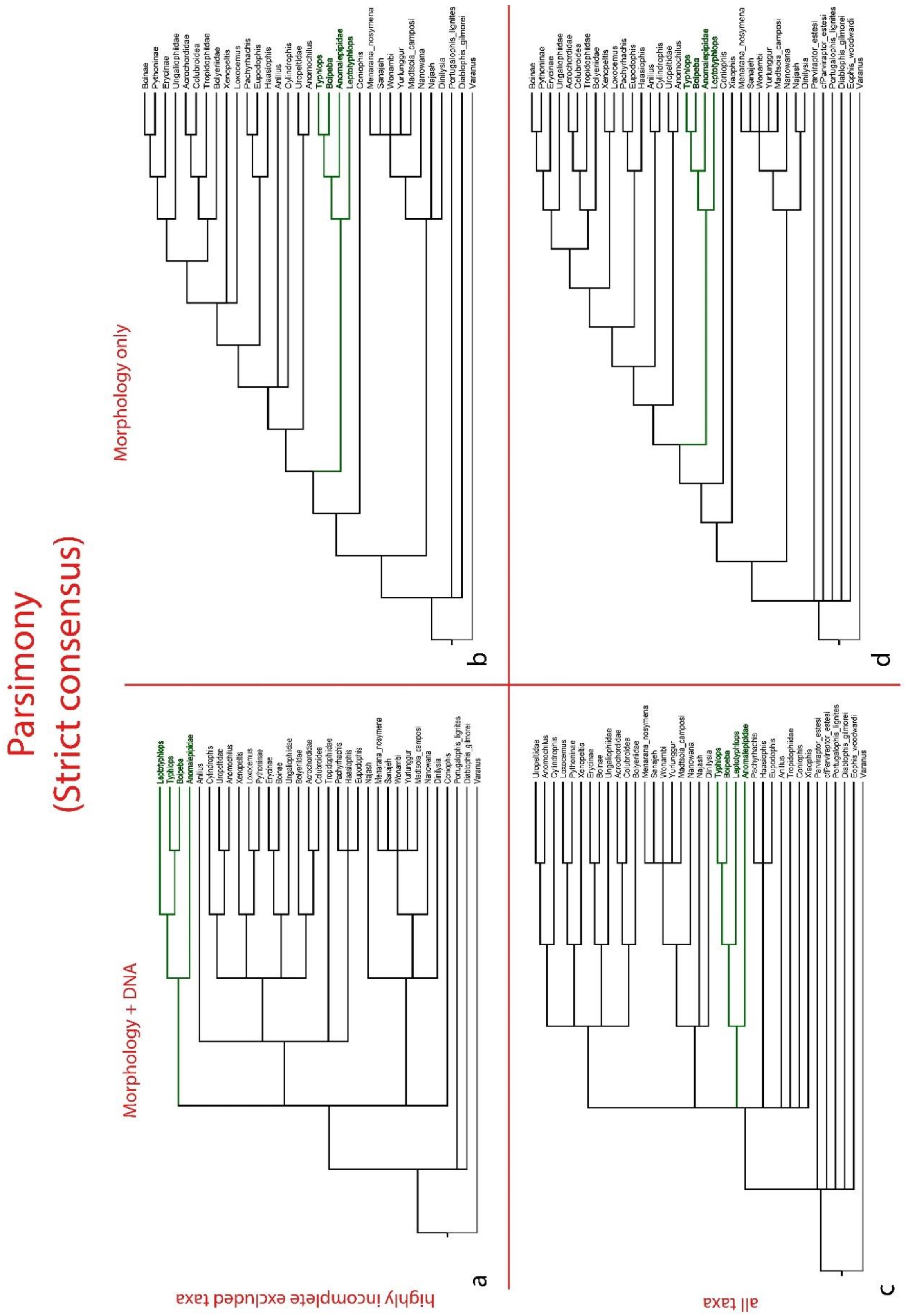

Fig. S4. Relationships of *Boipeba* and major snake lineages, based on parsimony analysis; strict consensus trees of shortest trees from PAUP. Related to Fig. 4. Scolecophidian (blindsnake) taxa in green. **a**. Morphology and DNA, with highly complete taxa excluded; **b**. Morphology only, with highly complete taxa excluded; **c**. Morphology and DNA (all taxa); and **d**. Morphology only (all taxa).

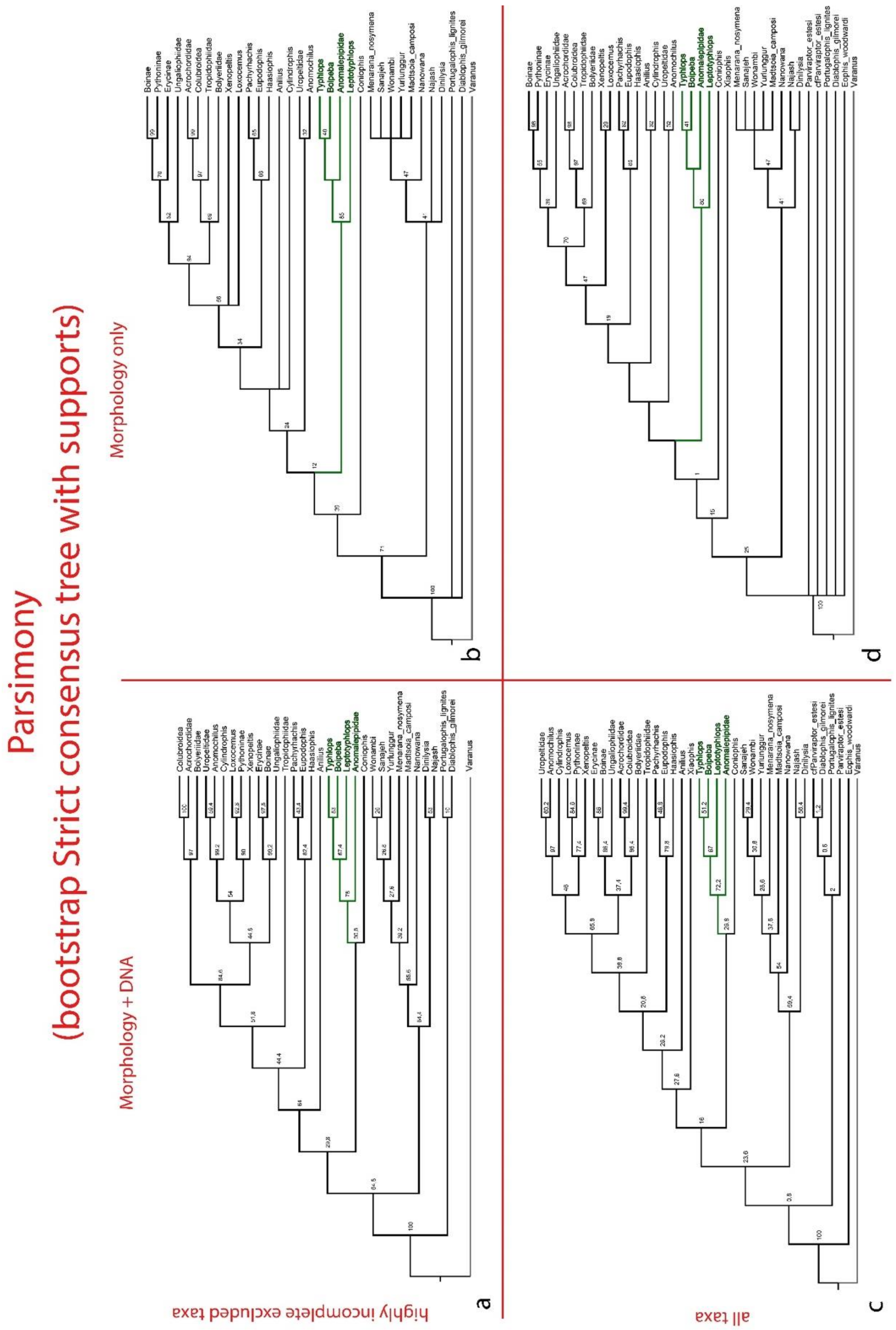

Fig. S5. Relationships of *Boipeba* and major snake lineages, based on parsimony analysis; majority-rule bootstrap consensus trees from TNT. Related to Fig. 4. Numbers denote bootstrap support for each clade. Scolecophidian (blindsnake) taxa in green. **a.** Morphology and DNA, with highly incomplete taxa excluded; **b.** Morphology only, with highly incomplete taxa excluded; **c.** Morphology and DNA (all taxa); **d.** Morphology only (all taxa).

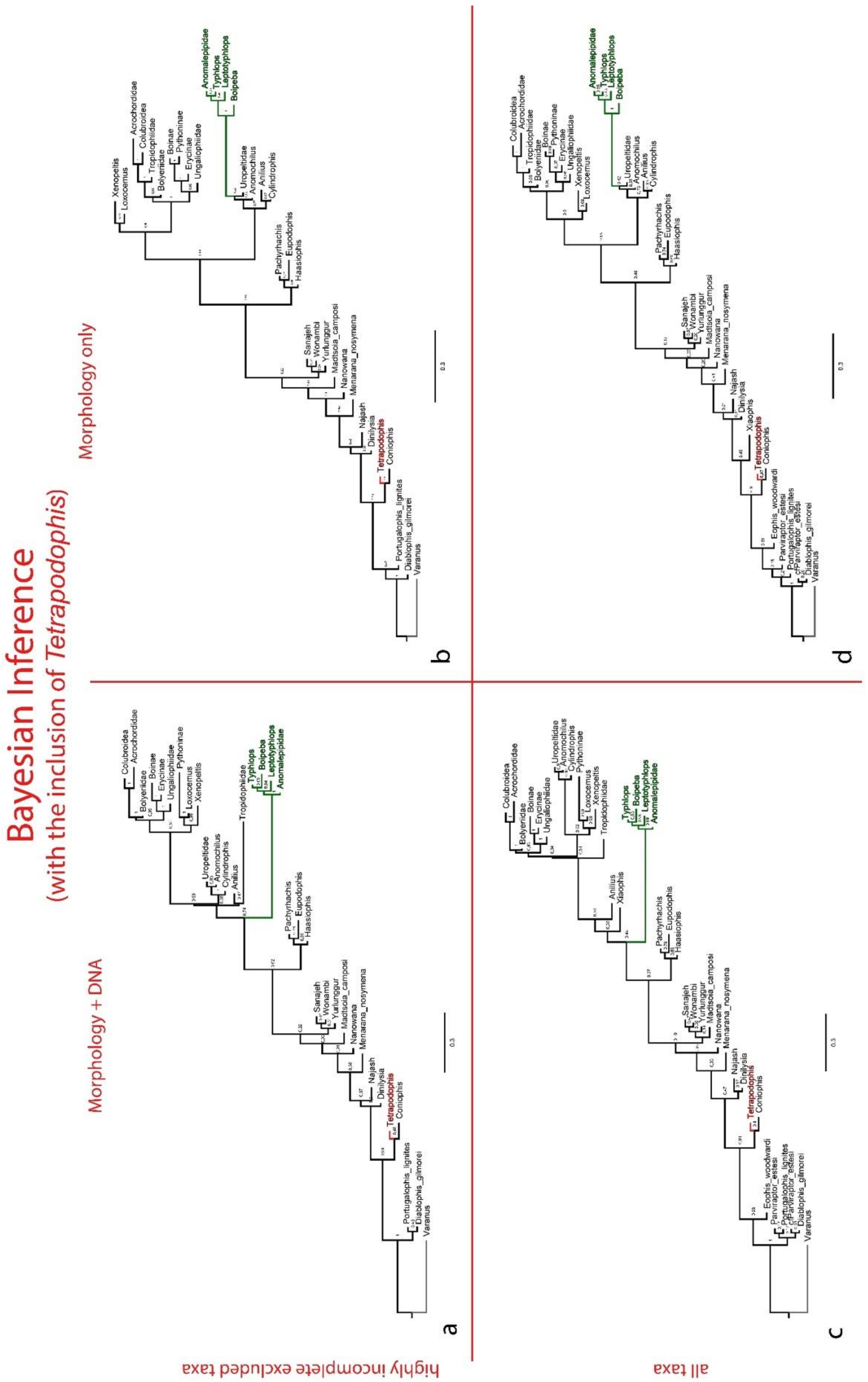

Fig. S6 Relationships of *Boipeba* and major snake lineages, based on Bayesian inference with the inclusion of the enigmatic and putative ophidian *Tetrapodophis amplectus*; majority-rule consensus trees from MrBayes. **Related to Fig. 4.** Numbers denote clade posterior probabilities. Scolecophidian (blindsnake) taxa in green. **a**. Morphology and DNA, with highly incomplete taxa excluded; **b**. Morphology only, with highly incomplete taxa excluded; **c**. Morphology and DNA (all taxa); **d**. Morphology only (all taxa)

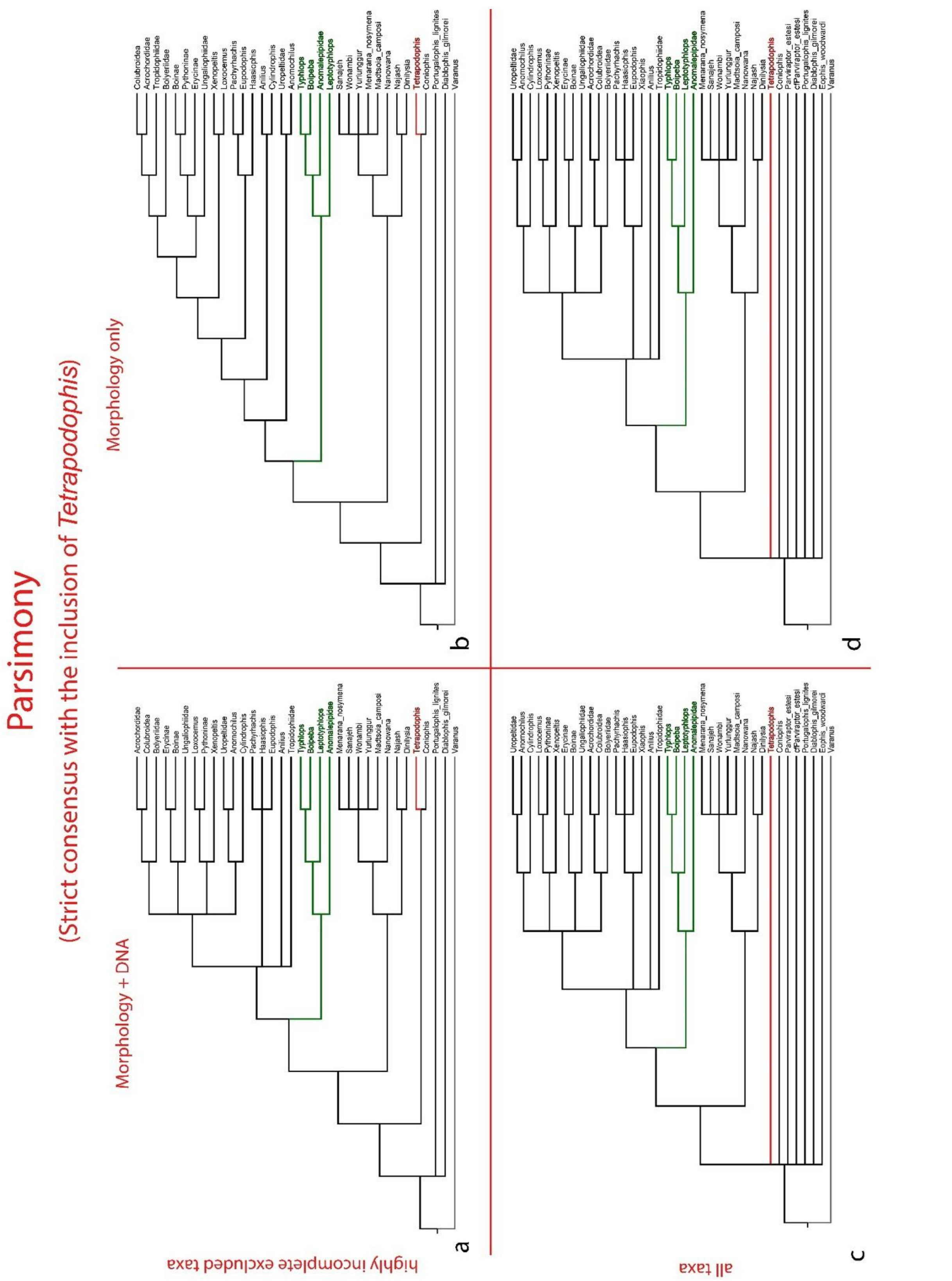

Fig. S7. Relationships of *Boipeba* and major snake lineages, based on parsimony analysis with the inclusion of the enigmatic and putative ophidian *Tetrapodophis amplectus*; strict consensus trees of shortest trees from PAUP. Related to Fig. 4. Scolecophidian (blindsnake) taxa in green. **a**. Morphology and DNA, with highly complete taxa excluded; **b**. Morphology only, with highly complete taxa excluded; **c**. Morphology and DNA (all taxa); and **d**. Morphology only (all taxa).

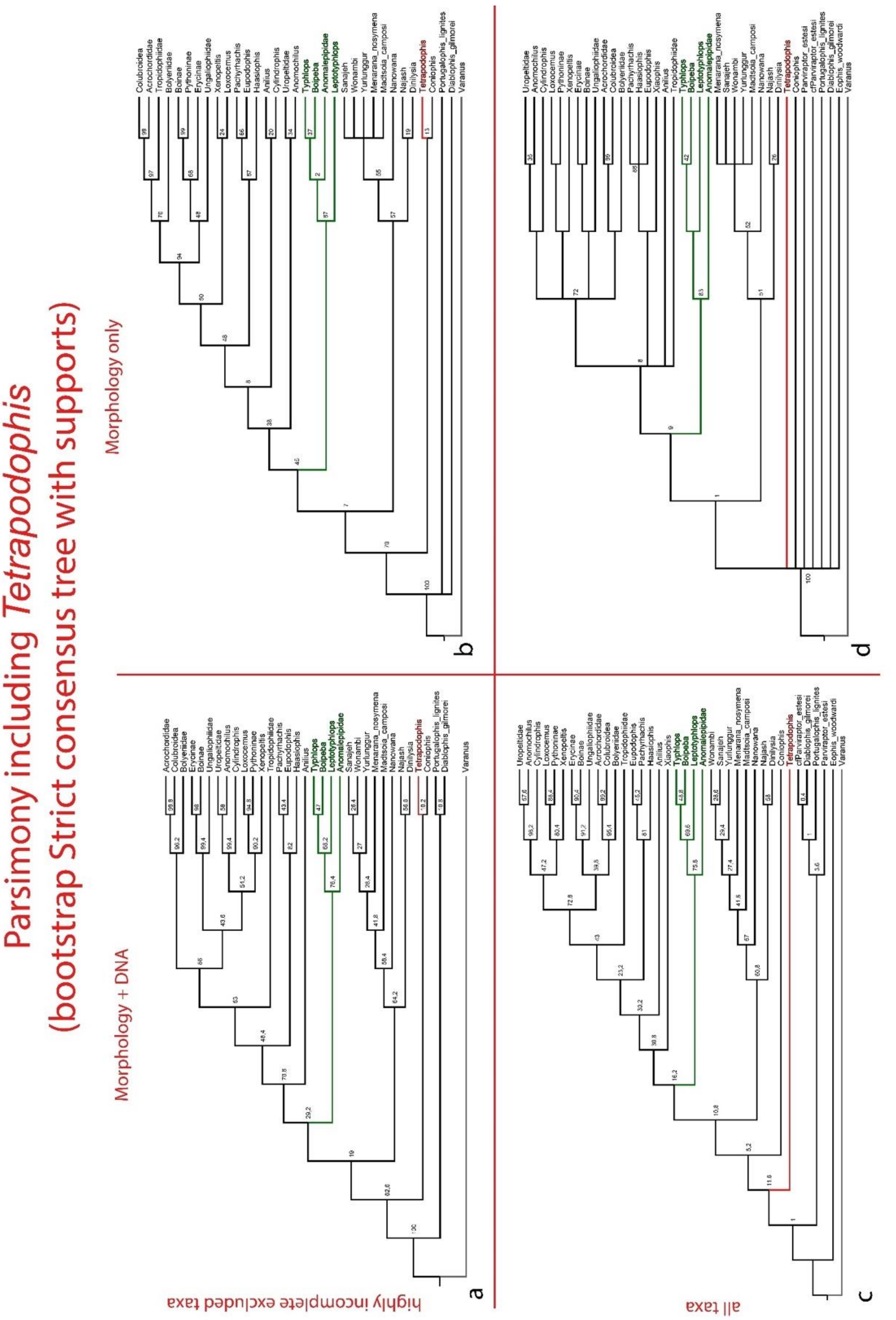

Fig. S8. Relationships of *Boipeba* and major snake lineages, based on parsimony analysis with the inclusion of the enigmatic putative ophidian *Tetrapodophis amplectus*; majority-rule bootstrap consensus trees from TNT. Related to Fig. 4. Numbers denote bootstrap support for each clade. Scolecophidian (blindsnake) taxa in green. **a.** Morphology and DNA, with highly incomplete taxa excluded; **b.** Morphology only, with highly incomplete taxa excluded; **c.** Morphology and DNA (all taxa); **d.** Morphology only (all taxa).

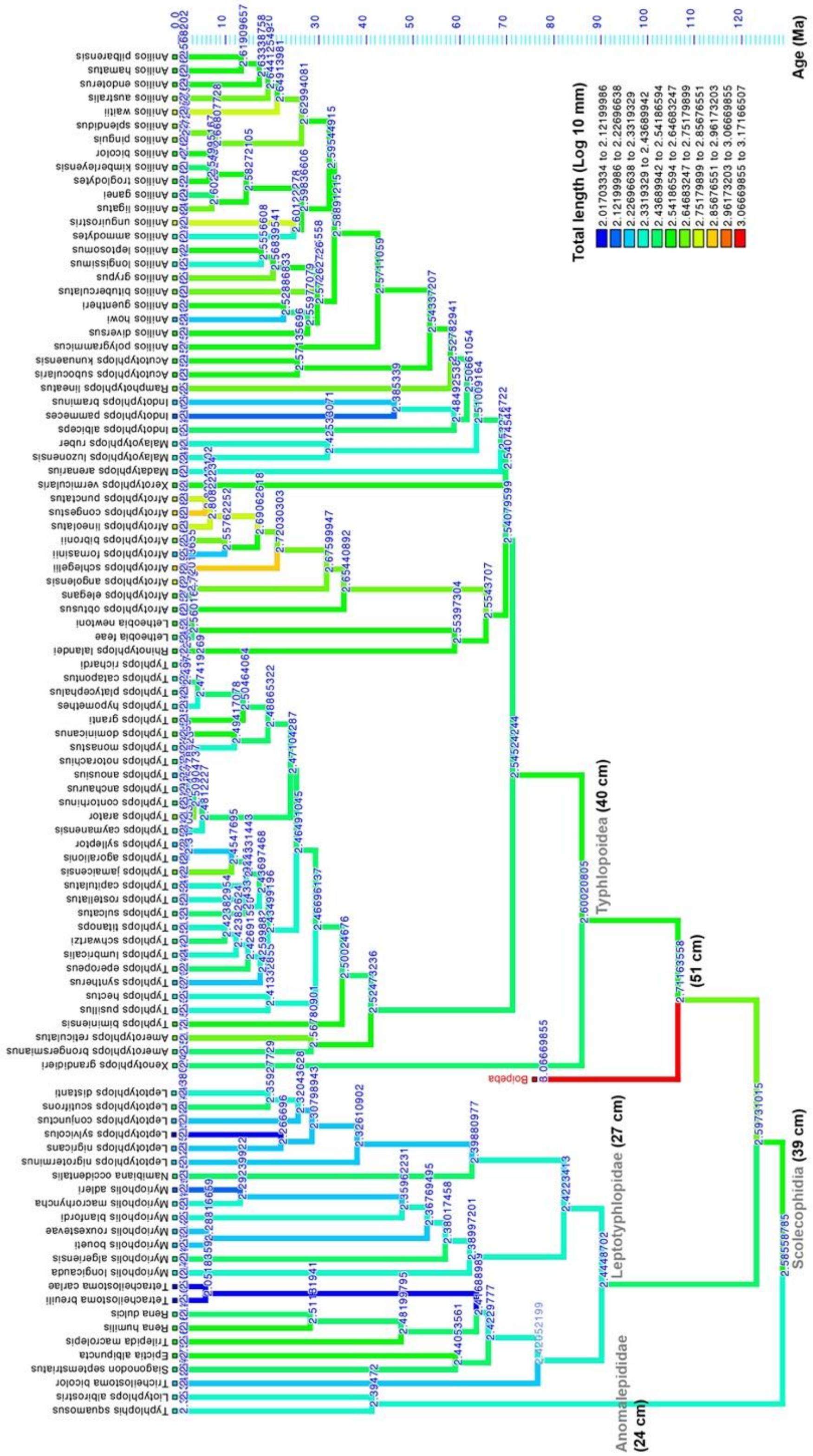

Fig. S9. Evolution of body size (total length) in scolecophidians. Related to Fig. 4. The analysis is based on squared-change parsimony with time-scaled branch lengths (=maximum likelihood with simple Brownian motion) in Mesquite. Warmer colours denote larger size. Numbers denote body length in mm (to log_{10} scale). For additional information consult Supplementary data 2.

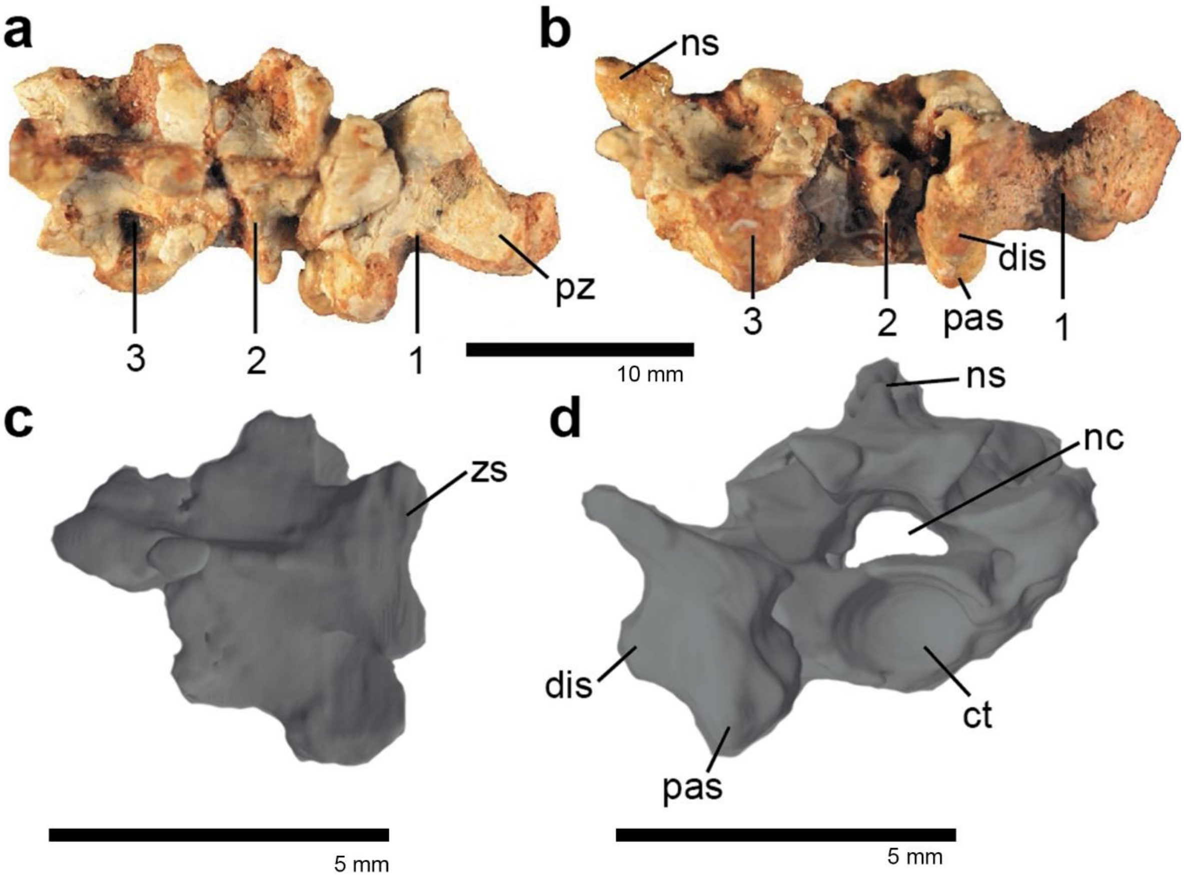

Figure S10. Set of three articulated vertebrae of indeterminate ophidian (unregistered specimen). **Related to Fig. 1. a** Dorsal view of the three articulated vertebrae (anterior to the right). **b** Lateral view (anterior to the right). **c** Dorsal view of digital reconstruction (i.e., segmented from micro CT) of posterior-most vertebra in the series (anterior to the right). **d** Anterior view of posterior-most vertebra in the series. Morphological differences from *Boipeba* include the concave anterior margin of the zygosphene, the distinctly trefoil-shaped cross section of the neural canal, and the presence of synapophyses subdivided into para- and diapophyseal facets. Abbreviations: dis, diapophyseal articular facet of the synapophysis; ct, cotyle; nc, neural canal; ns, neural spine; pas, parapophyseal articular facet of the synapophysis; pz, prezygapophysis; zs, zygosphene.
